# Association of a Mediterranean Diet With Outcomes for Patients Treated With Immune Checkpoint Blockade for Advanced Melanoma

**DOI:** 10.1001/jamaoncol.2022.7753

**Published:** 2023-02-16

**Authors:** Laura A. Bolte, Karla A. Lee, Johannes R. Björk, Emily R. Leeming, Marjo J. E. Campmans-Kuijpers, Jacco J. de Haan, Arnau Vich Vila, Andrew Maltez-Thomas, Nicola Segata, Ruth Board, Mark Harries, Paul Lorigan, Elisabeth G. E. de Vries, Paul Nathan, Rudolf Fehrmann, Véronique Bataille, Tim D. Spector, Geke A. P. Hospers, Rinse K. Weersma

**Affiliations:** 1 Department of Gastroenterology and Hepatology, University of Groningen and University Medical Center Groningen, Groningen, the Netherlands; 2 Department of Genetics, University of Groningen and University Medical Center Groningen, Groningen, the Netherlands; 3 Department of Twin Research and Genetic Epidemiology, King’s College London, England; 4 Department of Medical Oncology, University of Groningen and University Medical Center Groningen, Groningen, the Netherlands; 5 Department of Cellular, Computational and Integrative Biology, University of Trento, Trento, Italy; 6 European Institute of Oncology, IRCSS, Milan, Italy; 7 Department of Medical Oncology, Royal Preston Hospital, Lancashire National Health Service (NHS) Foundation Trust, Fulwood, England; 8 Department of Medical Oncology, Guys Cancer Centre, Guys, and St Thomas’s NHS Trust, London, England; 9 The Christie NHS Foundation Trust, Manchester, England; 10 Department of Medical Oncology, Mount Vernon Cancer Centre, Northwood, England; 11 Department of Dermatology, West Hertfordshire NHS Trust, England

## Abstract

**Importance:**

Immune checkpoint blockade (ICB) has improved the survival of patients with advanced melanoma. Durable responses are observed for 40% to 60% of patients, depending on treatment regimens. However, there is still large variability in the response to treatment with ICB, and patients experience a range of immune-related adverse events of differing severity. Nutrition, through its association with the immune system and gut microbiome, is a poorly explored but appealing target with potential to improve the efficacy and tolerability of ICB.

**Objective:**

To investigate the association between habitual diet and response to treatment with ICB.

**Design, Setting, and Participants:**

This multicenter cohort study (the PRIMM study) was conducted in cancer centers in the Netherlands and UK and included 91 ICB-naive patients with advanced melanoma who were receiving ICB between 2018 and 2021.

**Exposures:**

Patients were treated with anti–programmed cell death 1 and anti–cytotoxic T lymphocyte–associated antigen 4 monotherapy or combination therapy. Dietary intake was assessed through food frequency questionnaires before treatment.

**Main Outcomes and Measures:**

Clinical end points were defined as overall response rate (ORR), progression-free survival at 12 months (PFS-12), and immune-related adverse events that were grade 2 or higher.

**Results:**

There were a total of 44 Dutch participants (mean [SD] age, 59.43 [12.74] years; 22 women [50%]) and 47 British participants (mean [SD] age, 66.21 [16.63] years; 15 women [32%]). Dietary and clinical data were prospectively collected from 91 patients receiving ICB between 2018 and 2021 for advanced melanoma in the UK and the Netherlands. Logistic generalized additive models revealed positive linear associations between a Mediterranean dietary pattern that was high in whole grains, fish, nuts, fruit, and vegetables and the probability of ORR and PFS-12 (probability of 0.77 for ORR; *P* = .02; false discovery rate, 0.032; effective degrees of freedom, 0.83; probability of 0.74 for PFS-12; *P* = .01; false discovery rate, 0.021; effective degrees of freedom, 1.54).

**Conclusions and Relevance:**

This cohort study found a positive association between a Mediterranean diet, a widely recommended model of healthy eating, and response to treatment with ICB. Large prospective studies from different geographies are needed to confirm the findings and further elucidate the role of diet in the context of ICB.

## Key Points

**Question:**

Is a habitual diet associated with tumor response to immune checkpoint blockade (ICB) in advanced melanoma?

**Findings:**

In this cohort study of 91 patients with advanced melanoma in the UK and the Netherlands, higher adherence to the principles of a Mediterranean diet was associated with a higher probability of response to treatment with ICB.

**Meaning:**

The results of this study suggest that while further studies across different countries will be needed to confirm the findings and offer patient-specific advice, habitual diet may have a role in improving responses to ICB.

## Teaser

This cohort study examines the association between habitual diet and response to treatment with immune checkpoint blockade among patients with advanced melanoma.

## Introduction

While immune checkpoint blockade (ICB) has revolutionized the treatment of advanced melanoma, many patients do not tolerate and/or respond to this treatment.^1^ Recent evidence suggests that variability in the efficacy of ICB is partially explained by differences in the gut microbiome.^2^ The abundance of several gut bacteria predictive of response to ICB is associated with diet.^3^ For example, dietary fiber is degraded to short-chain fatty acids (SCFAs) by bacteria, such as bifidobacteria, and high fiber intake and fecal SCFA concentrations have been associated with response in mice and ICB-treated patients.^4,5,6^ While evidence supporting the immunomodulatory and antitumor activities of specific nutrients is increasing, to our knowledge, studies comprehensively assessing the association of overall diet composition with ICB response are still lacking. In this study, we aim to investigate associations between different dietary patterns and ICB response and immune-related adverse events (irAEs) using a multinational prospective cohort of patients with advanced melanoma.

## Methods

We prospectively collected dietary and clinical data from 91 patients who received ICB between 2018 and 2021 for advanced melanoma in the UK (PRIMM-UK) and the Netherlands (PRIMM-NL; eFigure 1 in Supplement 1). Participants provided written informed consent. PRIMM-UK (NCT03643289) was sponsored by East & North Hertfordshire National Health Service Trust with UK central ethical approval. PRIMM-NL was approved by the medical ethical committee of the University Medical Center Groningen in the Netherlands (POINTING NCT04193956; OncoLifeS METc 2010/109). Clinical end points were defined as overall response rate (ORR), progression-free survival at 12 months (PFS-12), and irAEs. Patients were classified as responders (complete response, partial response, or stable disease) or nonresponders (progressive disease) using the Response Evaluation Criteria in Solid Tumors, version 1.1. Immune-related adverse events were assessed using the Common Terminology Criteria for Adverse Events, version 5. As an outcome variable, we focused on irAEs grade 2 or higher to avoid the subjectivity and interindividual variability associated with the mildest of adverse events.

Dietary intake was assessed through the EPIC-Norfolk food frequency questionnaire^7^ and the Dutch Healthy Diet food frequency questionnaire^8^ (eMethods in Supplement 1). Food items were collapsed into standardized food groups using the national food composition databases (eTable 1 in Supplement 1). To account for differences in diets or nutritional profiling, we performed country-specific and joint analyses.

Four food-based scores were calculated to address dietary quality across cohorts (eTable 2 in Supplement 1) which were as follows: (1) alternate Mediterranean diet score (aMED)^9^; (2) original plant-based diet index (oPDI), which was further distinguished into^10^; (3) healthy plant-based diet index (h-PDI), and (4) unhealthy plant-based diet index (u-PDI). A principal component (PC) analysis was performed per cohort to identify data-driven dietary patterns. The first 5 PCs, collectively explaining 56.7% and 55.4% of total dietary variation in PRIMM-NL and PRIMM-UK, respectively, were retained for subsequent analyses (eFigures 2-3 in Supplement 1).

To determine whether a higher adherence to a particular diet is associated with a higher probability of response or irAEs, we used logistic generalized additive models.^11^ First, using the joint data set, we modeled ORR, PFS-12, or irAEs as outcome variable, and all 4 diet scores as independent variables while also adjusting for age, sex, body mass index (calculated as weight in kilograms divided by height in meters squared), and cohort. Next, we modeled each outcome variable and the first 5 PCs per cohort. To test which dietary pattern had the largest association with response and irAEs, we removed each diet score or PC from each model 1 at a time, keeping all other variables intact (eTable 3 in Supplement 1). Lastly, we analyzed specific food groups and nutrients in association with response and irAEs. Analyses were adjusted for multiple testing using the Benjamini-Hochberg method as implemented in the *p.adjust* function in R (R Foundation).

## Results

Cohort characteristics are summarized in the Table and eTable 4 in Supplement 1. The aMED had the largest association with ORR, PFS-12, and irAEs (explained deviance: 51%, 54%, and 24%, respectively; eTable 3 in Supplement 1). Both ORR and PFS-12 showed positive associations with the aMED score (Figure), for which the maximum score of 5 was associated with the highest probability of response (probability of 0.74 for PFS-12; *P* = .01; false discovery rate [FDR], .021; effective degrees of freedom [edf], 1.54; probability of 0.77 for ORR; *P* = .02; FDR*, *.032; edf, 0.83). The log odds of being a responder increased by 1.43 for every unit increase in the aMED score. We performed a cross prediction using the same model. Despite the limited number of samples in each cohort, training on PRIMM-UK and validating on PRIMM-NL was able to predict PFS-12 and ORR by the aMED score with an area under the curve of 0.70 (eFigure 2 in Supplement 1).

**Table.** Cohort Characteristics

Characteristic^a^	No. (%)		*P* value
	PRIMM-NL (n = 44)	PRIMM-UK (n = 47)	
Age at stage IV diagnosis, mean (SD), y	59.43 (12.74)	66.21 (16.63)	.02
BMI, mean (SD)	27.51 (5.55)	29.06 (5.32)	.19
Sex			
Female	22 (50)	15 (32)	.12
Male	22 (50)	32 (68)	
Outcomes following ICB			
PFS-12	20 (46)	23 (49)	.93
ORR	26 (59)	27 (58)	>.99
irAEs (CTCAE grade ≥2)	21 (48)	25 (53)	.76
Metastatic stage			
Stage 3, unresectable	1 (2)	4 (9)	.01^b^
M1a	6 (14)	11 (23)	
M1b	8 (18)	11 (23)	
M1c	12 (27)	17 (36)	
M1d	17 (39)	4 (9)	
*BRAF* variant	23 (52)	14 (30)	.049
ECOG performance score ≥1	16 (36)	33 (70)	.002
ICB regimen			
Ipilimumab-nivolumab combination	11 (25)	23 (49)	.04
Single agent			
PD-1/PD-L1 inhibition	32 (73)	24 (51)	
CTLA-4 inhibition	1 (2)	0 (0)	
Previous *BRAF* or MEK inhibition	17 (39)	9 (19)	.07
Antibiotic use at baseline	10 (23)	8 (18)	.71
PPI use at baseline	19 (43)	12 (26)	.12
Diet scores, mean (SD)			
aMED	3.07 (1.25)	2.55 (1.28)	.08
Original PDI	30.52 (4.29)	34.23 (4.45)	<.001^b^
hPDI	32.84 (5.81)	35.49 (7.37)	.13
uPDI	31.70 (4.56)	34.32 (5.65)	.02

Abbreviations: aMED, alternate Mediterranean diet score; BMI, body mass index (calculated as weight in kilograms divided by height in meters squared); *BRAF*, v-raf murine sarcoma viral oncogene homologue B1; CTCAE, Common Terminology Criteria for Adverse Events; CTLA-4, cytotoxic T lymphocyte–associated antigen 4; ECOG, Eastern Cooperative Oncology Group; hPDI, healthy plant-based diet index; ICB, immune checkpoint blockade; irAEs, immune-related adverse events; MEK, mitogen-activated protein kinase; NL, Netherlands; original PDI, original plant-based diet index; ORR, overall response rate; PD-1, programmed cell death 1; PD-L1, programmed cell death ligand 1; PFS-12, progression-free survival at 12 months; PPI, proton pump inhibitor; UK, United Kingdom; uPDI, unhealthy plant-based diet index.

^a^ Characteristics of the PRIMM cohorts. Baseline characteristics are presented as mean (SD) for continuous variables and counts and percentages for categorical variables. χ^2^ tests for categorical variables and the Mann-Whitney *U* test for continuous data were performed to calculate differences between cohorts.

^b^ Statistical significance with a false discovery rate of 5%.

**Figure.**
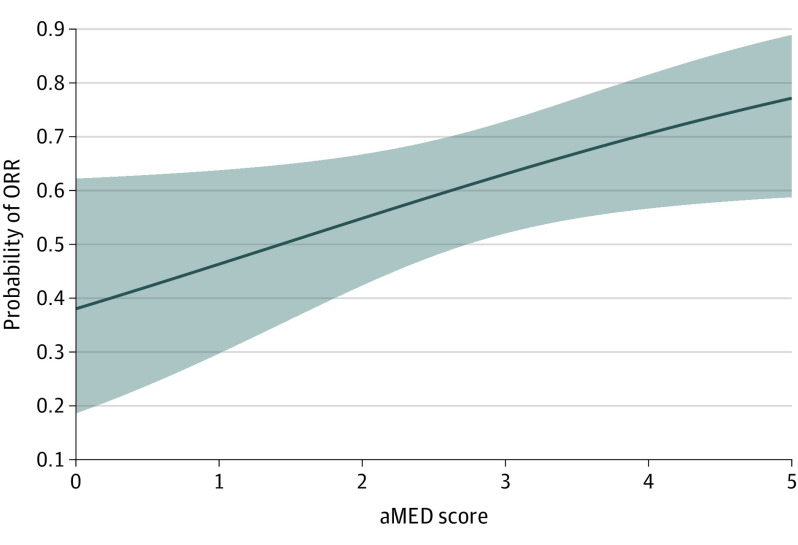
Association Between Overall Response Rate (ORR) and the Alternate Mediterranean Diet Score (aMED) Across Both Cohorts The y-axis shows the probability of ORR on a scale from 0 to 0.9. The x-axis shows adherence to a Mediterranean diet high in vegetables, legumes, fruit, and whole grains and low in red and processed meat, expressed by the aMED score ranging from 0 (minimum score) to 5 (maximum score).

The analysis of PCs per cohort revealed a parabolic association of PC2, which was characterized by a high fruit intake, with PFS-12 (*P* = .01; FDR, .018; edf, 2.14) and ORR in PRIMM-NL (*P* = .01; FDR, .018; edf, 2.7). No significant associations were found for PRIMM-UK (eTable 7 and eFigures 2-5 in Supplement 1).

Individual components of the Mediterranean diet, including monounsaturated and polyunsaturated fatty acids, whole-meal bread, vegetables, legumes, and potatoes, followed a similar positive association with response as the aMED score, as did vitamins C and E and β-carotene. However, these associations were not significant after multiple hypothesis testing correction. Similarly, we observed fewer irAEs with high legume, whole-meal bread, and magnesium consumption but more irAEs with higher processed meat intake (eFigure 6 and eTables 8-9 in Supplement 1).

## Discussion

In this cohort study, we examined dietary patterns in association with response to treatment with ICB across patients from the UK and the Netherlands. The results suggested that a Mediterranean-style diet that is enriched in whole grains, fish, nuts, fruit, legumes and vegetables is associated with a higher probability of response in ICB-treated patients with advanced melanoma.

The traditional principles of the Mediterranean diet (ie, high in plant-derived foods and low in processed foods and meat) remain the most widely used dietary recommendations of public health institutions globally. A potential mechanism underlying the association between diet and immunotherapy response is the gut microbiome. Preclinical studies have shown immunomodulatory and antitumor activities of several nutrients, including fiber, polyphenols, and antioxidants, that are mediated via the gut microbiome (eTable 10 in Supplement 1).^12,13,14,15^ The Mediterranean diet has been associated with an increased abundance of bacteria producing SCFAs^3^ that have been shown to be predictive of immunotherapy response in several studies.^2,4,5,6^ Within a published cohort of 52 patients treated with ICB for different solid tumors, high fecal SCFA concentrations were shown to be associated with longer PFS.^6^

There are 2 reports that describe an association between specific nutrients (fiber^5,12^ and omega-3 fatty acids^12^) and ICB response in patients. In a cohort of 128 patients with melanoma, patients reporting a high fiber intake were more likely to respond, which was confirmed in conventionally housed specific pathogen–free mice, but not in germ-free mice, suggesting the gut microbiome as a mediator. A study in patients with stage 3 melanoma found omega-3 fatty acids to be associated with response and butyrate-producing microbial pathways. The Mediterranean diet is characterized by a high content of fiber from plant-derived foods and unsaturated fats from fish and nuts; as such, these studies support our findings.^5,6,12^

### Strengths and Limitations

Collecting extensive dietary data from patients with advanced cancer is challenging, and the primary strengths of this study are the prospective dietary assessment and depth of data collected from a real-world population of patients across 2 European countries. Limitations included sample size and the difference between UK and Dutch food frequency questionnaires. To overcome these limitations, we have (1) accounted for differences in the statistical models used and (2) chosen to complement the analysis of foods and nutrients by diet scores that are comparable across countries. Specific food preferences and nutrient sources vary across geographies,^5,6,12^ suggesting a need for multinational cohort studies paired with more resolution on food compositions.

## Conclusions

The results of this cohort study suggest that the Mediterranean dietary pattern is associated with a higher probability of PFS and ORR in patients receiving ICB for advanced melanoma. These findings suggest a potential role for diet in improving responses to ICB treatment outcomes.

## Notes

Supplement 1. eMethods. eFigure 1. Flowchart depicting the design of the PRIMM-study eFigure 2. Results of the cross-prediction analysis eFigure 3. Principal component analysis (PCA) per cohort eFigure 4. Dietary patterns derived from PCA eFigure 5. Relationships between the probability of response and PCA-derived dietary patterns eFigure 6. Relationships between treatment outcomes and specific nutrients and food groups eTable 1. Composition of food groups eTable 2. Composition of dietary scores eTable 3. Backward selection for variable importance eTable 4. Descriptive statistics eTable 5. Results of the generalized additive models using diet scores eTable 6. Loadings of food groups per dietary pattern identified by PCA and their correlations eTable 7. Results of the generalized additive models using the top 5 diet PCs eTable 8. Results of the generalized additive models using nutrients eTable 9. Results of the generalized additive models using individual food groups eTable 10. Overview of literature linking cancer, nutrition, and immune checkpoint blockade eTable 11. Description of outliers that corresponded to an implausible consumption References.

Supplement 2. Data sharing statement
