# Supplementary material for: Association of a Mediterranean Diet With Outcomes for Patients Treated With Immune Checkpoint Blockade for Advanced Melanoma
Source: JAMA Oncol. 2023 Feb 16;9(5):705–9. doi: 10.1001/jamaoncol.2022.7753 (PMC9936383; doi:10.1001/jamaoncol.2022.7753)
Supplement: Supplement 1. — eMethods. eFigure 1. Flowchart depicting the design of the PRIMM-study eFigure 2. Results of the cross-prediction analysis eFigure 3. Principal component analysis (PCA) per cohort eFigure 4. Dietary patterns derived from PCA eFigure 5. Relationships between the probability of response and PCA-derived dietary patterns eFigure 6. Relationships between treatment outcomes and specific nutrients and food groups eTable 1. Composition of food groups eTable 2. Composition of dietary scores eTable 3. Backward selection for variable importance eTable 4. Descriptive statistics eTable 5. Results of the generalized additive models using diet scores eTable 6. Loadings of food groups per dietary pattern identified by PCA and their correlations eTable 7. Results of the generalized additive models using the top 5 diet PCs eTable 8. Results of the generalized additive models using nutrients eTable 9. Results of the generalized additive models using individual food groups eTable 10. Overview of literature linking cancer, nutrition, and immune checkpoint blockade eTable 11. Description of outliers that corresponded to an implausible consumption References. [file jamaoncol-e227753-s001.pdf]

# Supplemental Online Content

Bolte LA, Lee KA, Björk JR, et al. Association of a Mediterranean diet with outcomes for patients treated with immune checkpoint blockade for advanced melanoma. *JAMA Oncology*. Published online February 16, 2023.
doi:10.1001/jamaoncol.2022.7753

**eMethods.**
**eFigure 1.** Flowchart depicting the design of the PRIMM-study
**eFigure 2.** Results of the cross-prediction analysis
**eFigure 3.** Principal component analysis (PCA) per cohort
**eFigure 4.** Dietary patterns derived from PCA
**eFigure 5.** Relationships between the probability of response and PCA-derived dietary patterns
**eFigure 6.** Relationships between treatment outcomes and specific nutrients and food groups
**eTable 1.** Composition of food groups
**eTable 2.** Composition of dietary scores
**eTable 3.** Backward selection for variable importance
**eTable 4.** Descriptive statistics
**eTable 5.** Results of the generalized additive models using diet scores
**eTable 6.** Loadings of food groups per dietary pattern identified by PCA and their correlations
**eTable 7.** Results of the generalized additive models using the top 5 diet PCs
**eTable 8.** Results of the generalized additive models using nutrients
**eTable 9.** Results of the generalized additive models using individual food groups
**eTable 10.** Overview of literature linking cancer, nutrition, and immune checkpoint blockade
**eTable 11.** Description of outliers that corresponded to an implausible consumption
**eReferences.**

This supplemental material has been provided by the authors to give readers additional information about their work.

© 2023 Bolte LA et al. *JAMA Oncology*.

## eMethods

## Dietary assessment

We prospectively collected FFQs from 91 patients receiving ICB between 2018 and 2020 for advanced melanoma treated in the UK (EPIC-Norfolk FFQ) and the Netherlands (Dutch Healthy Diet-FFQ, DHD-FFQ), validated for the UK and Dutch populations, respectively, using standardized methods (1,2).

EPIC-Norfolk FFQs were transformed into nutrients and food intake in frequencies and grams per day using the corresponding FFQ EPIC tool for analysis (FETA) software (3). The DHD-FFQ was developed by the division of Human Nutrition of Wageningen University, the Netherlands, to assess habitual food intake in clinical practice but was not designed to estimate nutrient intake (2). To account for differences in nutritional profiling and differences in diets in the Netherlands and the UK (**eTable 1, eTable 4**), we performed both country-specific and joint analyses.

A ratio of energy intake over basal metabolic rate (EI:BMR) and macronutrient intake two standard deviations from the mean were considered to represent under- and over-reporting (4). Prior to performing association analyses, we removed any observation that corresponded to an implausible consumption (**eTable 11**).

Single food items from the EPIC-Norfolk-FFQ and DHD-FFQ were collapsed into 23 and 17 standardized food groups, respectively, using the national food composition databases (5,6) (**eTable 1**). We used published quantile-based scoring systems on food frequencies per day to pool the datasets and to perform a joint analysis across patients from the UK and the Netherlands (**eTable S2**). A data-driven, unsupervised dietary pattern analysis (Principal component analysis, PCA) was performed on food groups to identify country-specific dietary patterns (7).

### Diet scores

Four food-based scores were calculated to address dietary quality across cohorts:

- **alternate Mediterranean diet score (aMED)** (8), reflecting the principals of traditional Mediterranean diet i.e., high in vegetables, legumes, nuts, fruits, whole grains, fish, and low in red and processed meat.
- **original plant-based diet index (oPDI)** (9,10), reflecting a higher intake of plant-derived foods relative to animal-derived foods; further distinguished into:
- **healthy plant-based diet index (h-PDI),** reflecting high-quality, unprocessed plant-based foods; and the:
- **unhealthy plant-based diet index (u-PDI),** reflecting unhealthy plant-derived foods such as juice and refined grains

The composition of diet scores is given in **eTable 2**. To allow for a better comparison, we calculated modified scores, using only FFQ food groups shared across the two cohorts. In a complementary analysis, we also tested the effect of the full dietary scores in each subset.

### Principal component analysis per cohort

PCA was performed per cohort on standardized food groups to extract data-driven diet patterns. The first 5 principal components (PCs), collectively explaining 56.7% and 55.4% of total dietary variation in PRIMM-NL and PRIMM-UK, respectively, were retained for subsequent analyses (**eFigures 2-3, eTable S6**).

© 2023 Bolte LA et al. *JAMA Oncology.*

## Logistic generalized additive models (GAMS)

To determine whether a higher adherence to a particular diet is associated with a higher probability of response or irAEs, we used logistic generalized additive models (GAMs, 11). We used logistic GAMs for two main reasons: (1) diet scores (modeled as independent variables here) are ordinal; (2) Logistic GAMs provide a flexible method for revealing potentially non-linear relationships between categorical response variables and ordinal or continuous independent variables.

The reported P-values test the null hypothesis that the relationship is a flat line (1). The effective degrees of freedom (edf) represent the degree of which the focal relationship is (non-) linear. More specifically, an edf=1 indicates a perfectly linear relationship while any deviation from 1 indicates a non-linear relationship. We also report deviance explained which is a measure of goodness-of-fit for nonlinear models and is analogous to the unadjusted R2 for linear models. A logistic GAM similar to a logistic linear regression uses the logit function as the link function which is used to model the probability of "success" (here e.g., response) as a function of the independent variables included in the model. The logit function converts a linear combination of the independent variables to the scale of a probability (i.e., between 0 and 1). The logit link function is defined as $\text{logit}(p_i) = ln(\frac{p_i}{1-p_i})$ where $p_i$ is the probability for sample unit $i$, the $\frac{p_i}{1-p_i}$ is the odds of success, and the logit of $p_i$ is then the corresponding log-odds of success. Importantly, we can later reverse the transformation to calculate the probability of success as $p_i = \frac{odds}{1+odds}$. Using the logit link function $E(Y_i) = ln(\frac{p_i}{1-p_i})$, we constructed the below logistic GAMs. For succinctness, we denote this link function as $g(\cdot)$. We performed three sets of logistic GAMs.

We first fitted a joint model for both cohorts using all 4 diet scores as the independent variables also controlling for BMI, age, sex and cohort.

$$g(\cdot) = f_1(\text{aMED}) + f_2(\text{oPDI}) + f_3(\text{hPDI}) + f_4(\text{uPDI}) + f_5(\text{BMI}) + f_6(\text{age}) + f_7(\text{sex}) + f_8(\text{cohort}) \qquad (eq.1)$$

Then we fitted a separate model for each cohort, including the first 5 diet PCs as the independent variables also controlling for BMI, age and sex.

$$g(\cdot) = f_1(\text{PC1}) + f_2(\text{PC2}) + f_3(\text{PC3}) + f_4(\text{PC4}) + f_5(\text{PC5}) + f_6(\text{BMI}) + f_7(\text{age}) + f_8(\text{sex}) \qquad (eq.2)$$

Finally, in a sub-analysis performed in each cohort separately, we fitted models where we included either one of 30 nutrients or one of 23 food groups in PRIMM-UK and 17 food groups in PRIMM-NL (**eTable 1**). Nutrients and total calories were only available for the U.K. cohort. Because including all food groups or nutrients as independent variables in the same model will result in more coefficients than observations, we modelled each food group individually. While interesting relationships appear in this sub-analysis, it is likely that we are limited in statistical power.

$$g(\cdot) = f_1(\text{food group}) + f_2(\text{caloric intake}) + f_3(\text{BMI}) + f_4(\text{age}) + f_4(\text{sex}) \qquad (eq.3)$$

$$g(\cdot) = f_1(\text{nutrient}) + f_2(\text{caloric intake}) + f_3(\text{BMI}) + f_4(\text{age}) + f_4(\text{sex}) \qquad (eq.4)$$

In the above equations, $f$ denotes a smooth function which captures the impact of any independent variable, and depending on the underlying patterns in the data, these can be either linear or nonlinear.

**Backward selection for variable importance**

To test which dietary pattern had the largest influence on response and irAEs, we removed each diet score or PC from each model one at a time, keeping all other variables intact. Removing a dietary pattern with a low explanatory power will do little to the deviance explained, while removing a dietary pattern with a large explanatory power will substantially reduce the deviance explained by the focal model (**eTable 3**).

© 2023 Bolte LA et al. *JAMA Oncology*.

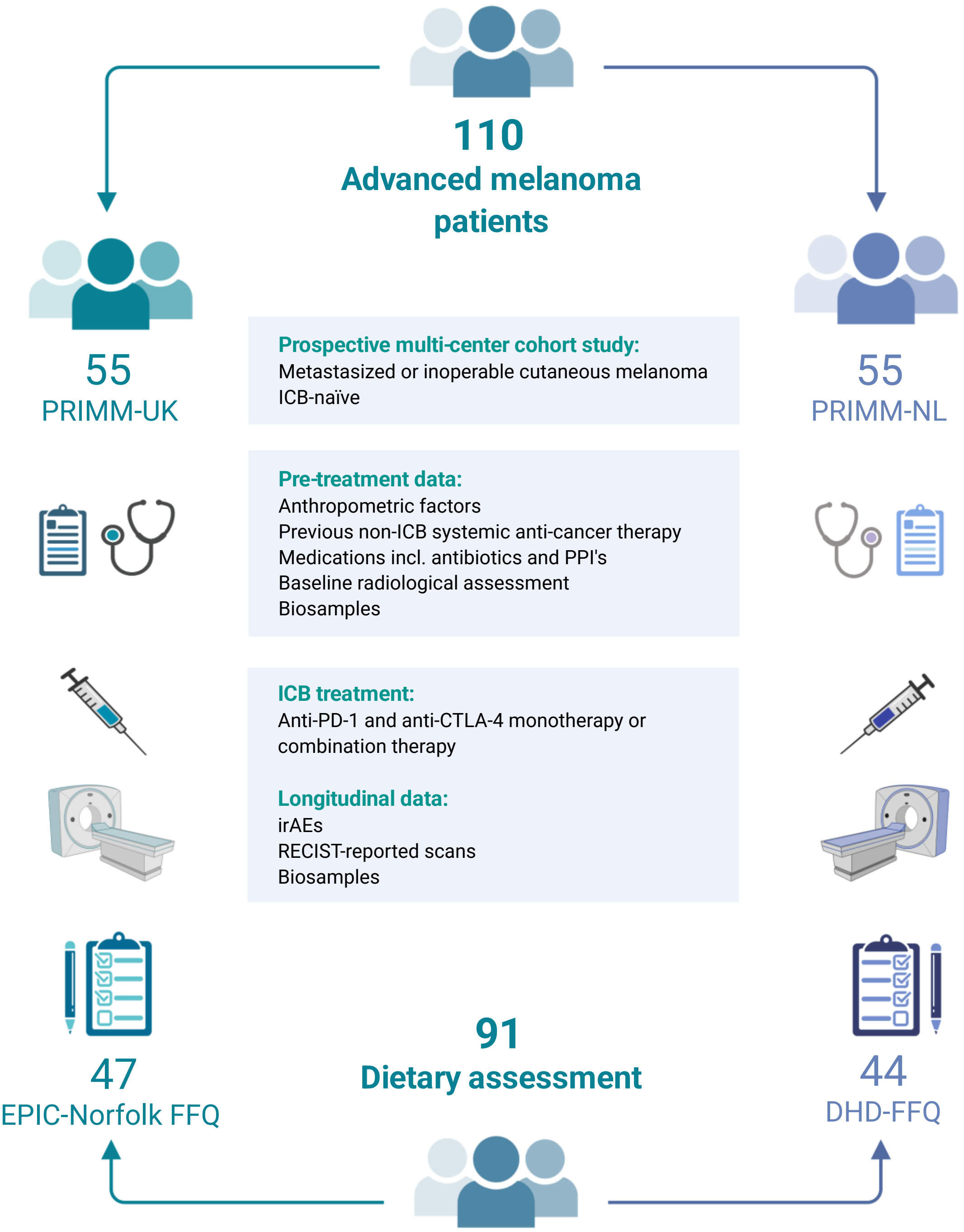


**eFigure 1. Flow chart depicting the design of the *PRIMM* study.** One hundred ten patients with advanced melanoma were treated with immune checkpoint blockade (ICB) at cancer centers in the UK and the Netherlands. All patients underwent response evaluation and provided extensive biosamples at regular intervals. In 91 patients (47 in the UK and 44 in the Netherlands), food frequency questionnaires and other pre-treatment data, including medication use, anthropometric factors, and tumor characteristics, were available. Abbreviations: PRIMM, Predicting Response to Immunotherapy for Melanoma with Gut Microbiome and Metabolomics; ICB, immune checkpoint blockade; PPI's, proton pump inhibitors; EPIC-Norfolk, European Prospective Investigation into Cancer; FFQ, food frequency questionnaire; DHD-FFQ, Dutch healthy diet food frequency questionnaire; RECIST, response evaluation criteria in solid tumors; irAEs, immune-related adverse events.

© 2023 Bolte LA et al. *JAMA Oncology*.

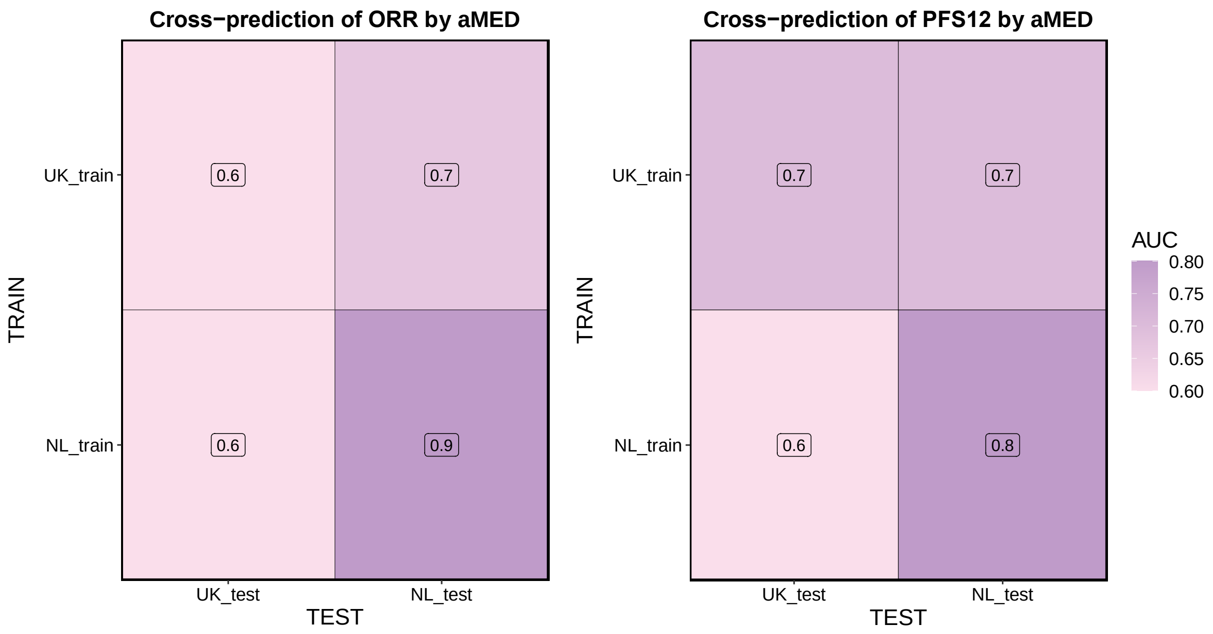


**eFigure 2. Results of the cross-prediction analysis.** Heatmap representing the predictive performance (AUC values) of the Mediterranean diet score when training (rows) and testing (columns) in the same cohort and when performing cross-predictions. High AUC values are indicated by the color density. Abbreviations: PFS12, progression-free survival at 12 months; ORR, Overall response rate; aMED, alternate Mediterranean diet score; AUC, Area under the curve; UK, United Kingdom; NL, Netherlands.

© 2023 Bolte LA et al. *JAMA Oncology*.

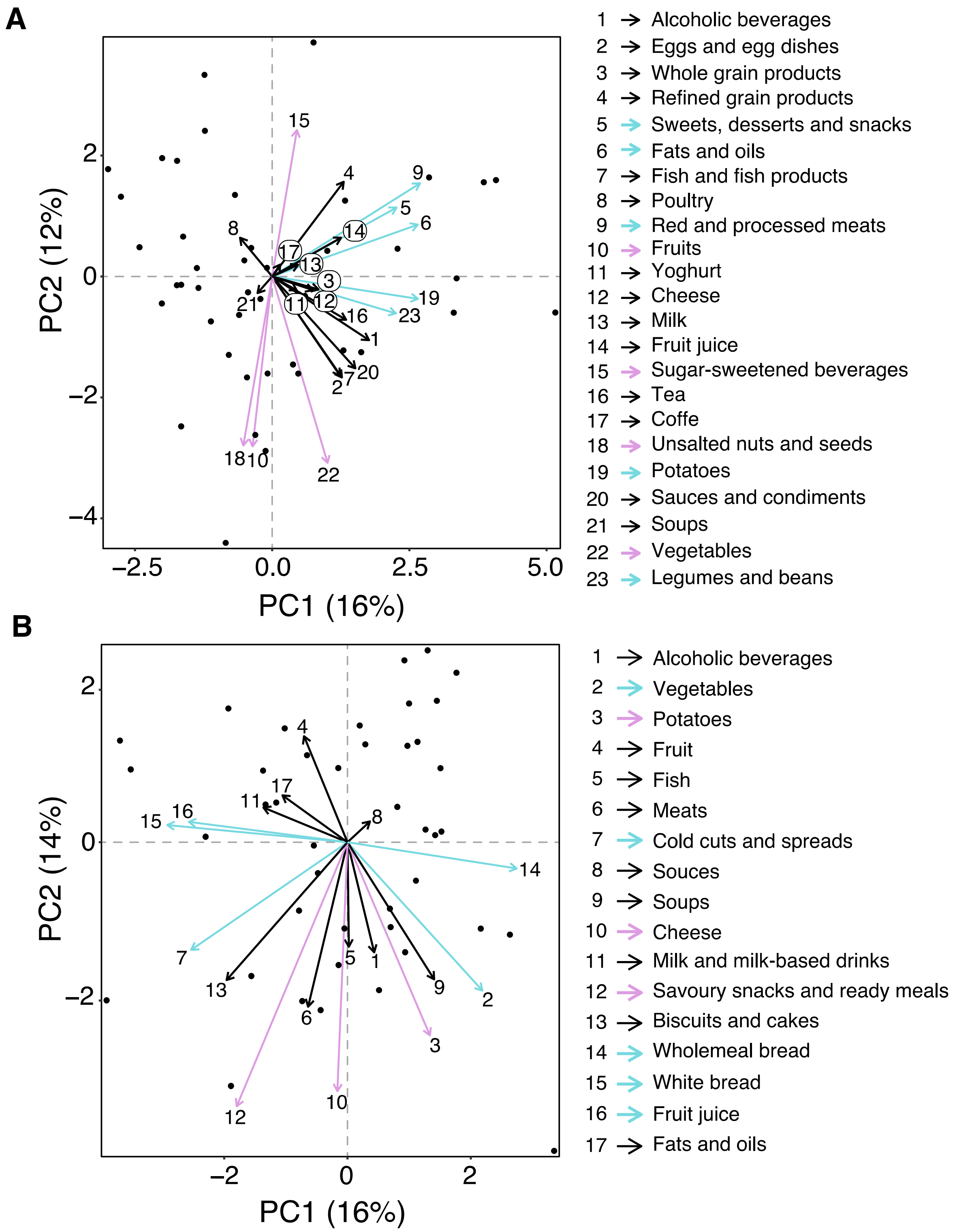


**eFigure 3. Principal Component Analysis (PCA) per cohort.** (A) PCA performed on 23 food groups in the PRIMM-UK cohort and (B) on 17 food groups in the PRIMM-NL cohort revealed a split in healthy and less healthy diets. While dots represent individual subjects, the length of the arrows indicates the size of the Pearson correlation coefficient. Colored arrows indicate food groups whose correlations with PC1 (teal) and with PC2 (pink) exceed 0.5. Abbreviations: PC, principal component.

© 2023 Bolte LA et al. *JAMA Oncology*.

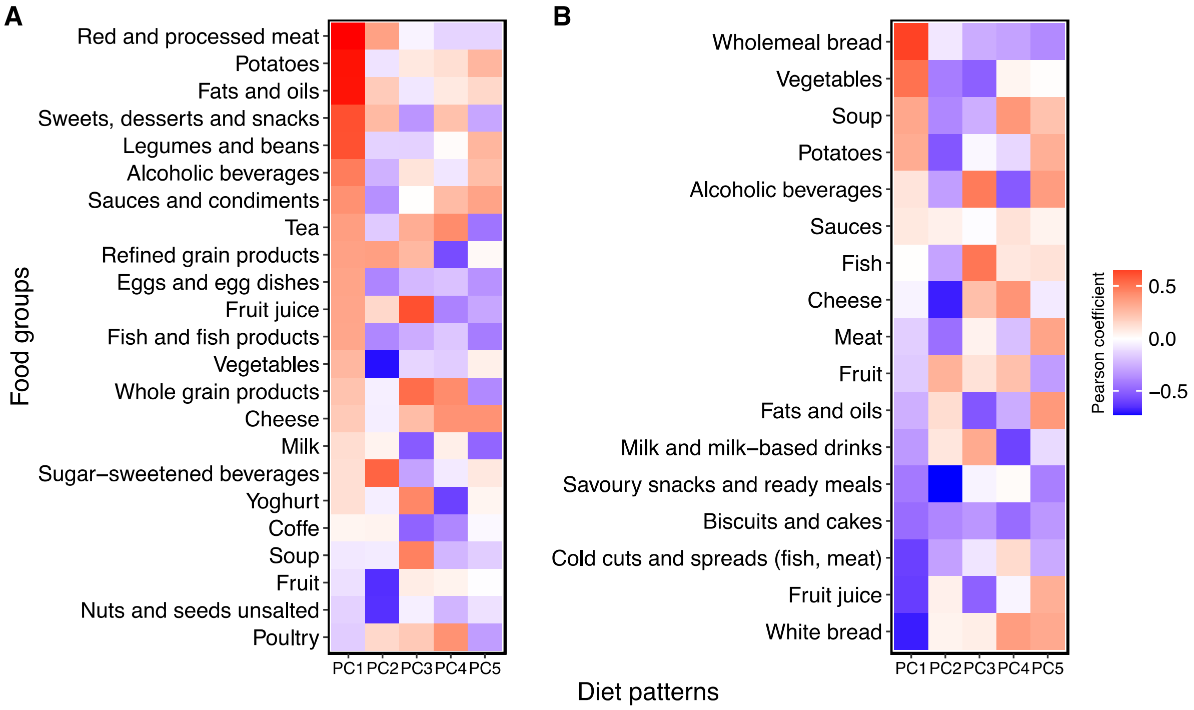


**eFigure 4. Dietary patterns derived from PCA.** (A) PRIMM-UK and (B) PRIMM-NL. Heatmap showing the Pearson correlation coefficient for each food group with the first five principal components (i.e., diet patterns). The y-axis shows cohort-specific food groups, ordered by PC1 in each cohort. Cells in red and blue indicate positive and negative correlations, respectively. The color density corresponds to the size of the correlation coefficient (see **eTable 6**). PC, principal component; PCA, principal component analysis.

© 2023 Bolte LA et al. *JAMA Oncology*.

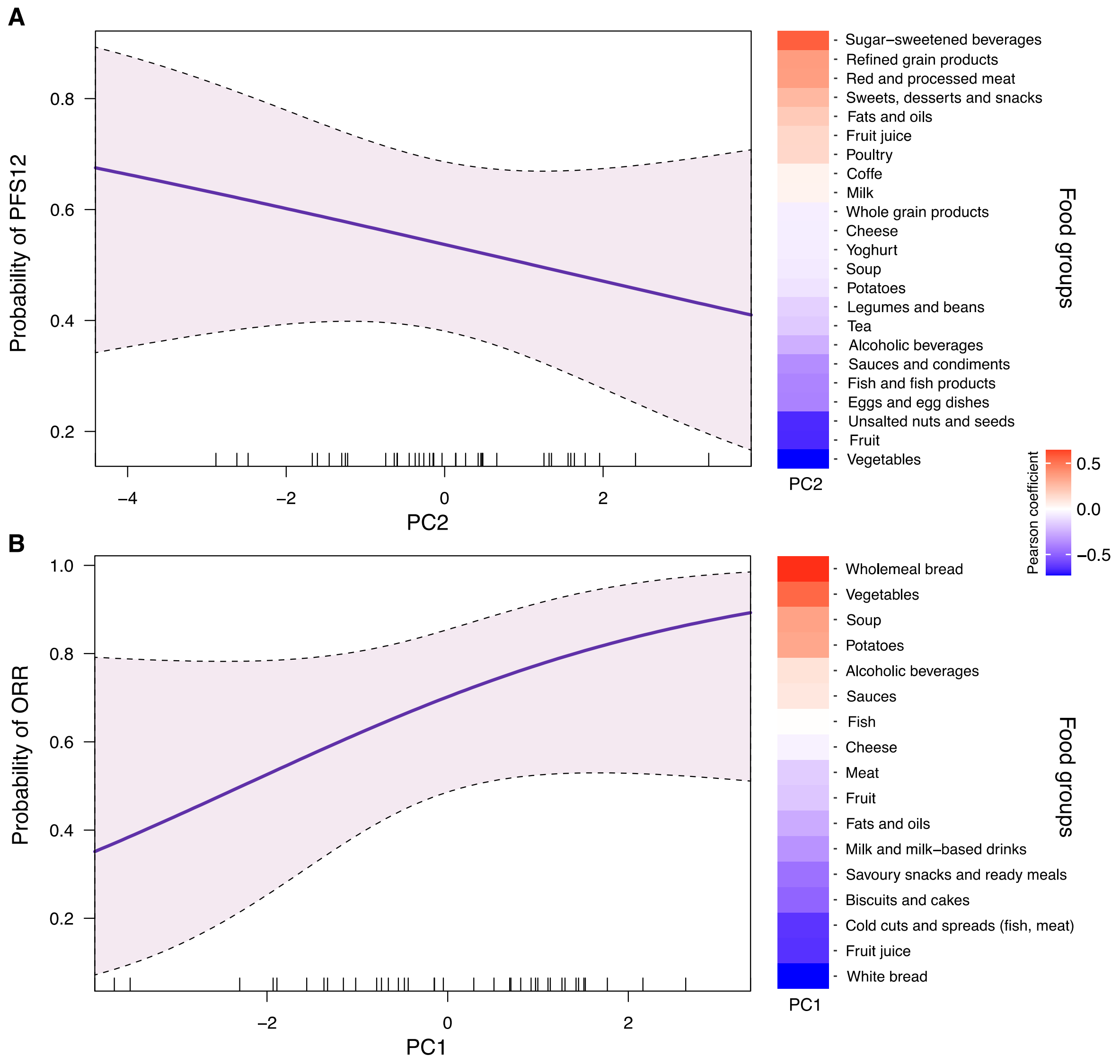


**eFigure 5. Relationships between the probability of response and PCA-derived dietary patterns.** (A) PC2 and PFS-12 in PRIMM-UK (B) PC1 and ORR in PRIMM-NL. The shaded area shows the 95% confidence intervals (CIs). Data-driven dietary pattern analysis was performed using PCA in each cohort. PC1 and PC2 described most of the dietary variation in each cohort. Panel A shows a negative relationship between PFS-12 and PC2, a Western dietary pattern, in the UK cohort. (B) Panel B illustrates a positive relationship between ORR and PC1, characterized by high intake of whole meal bread, vegetables and potatoes and a low intake of high-sugar foods in the Dutch cohort. The relationships were not significant after multiple hypothesis testing correction (*FDR* >.05, **eTable 7**). PFS-12, progression-free survival at 12 months; ORR, overall response rate; PC, principal component.

© 2023 Bolte LA et al. *JAMA Oncology*.

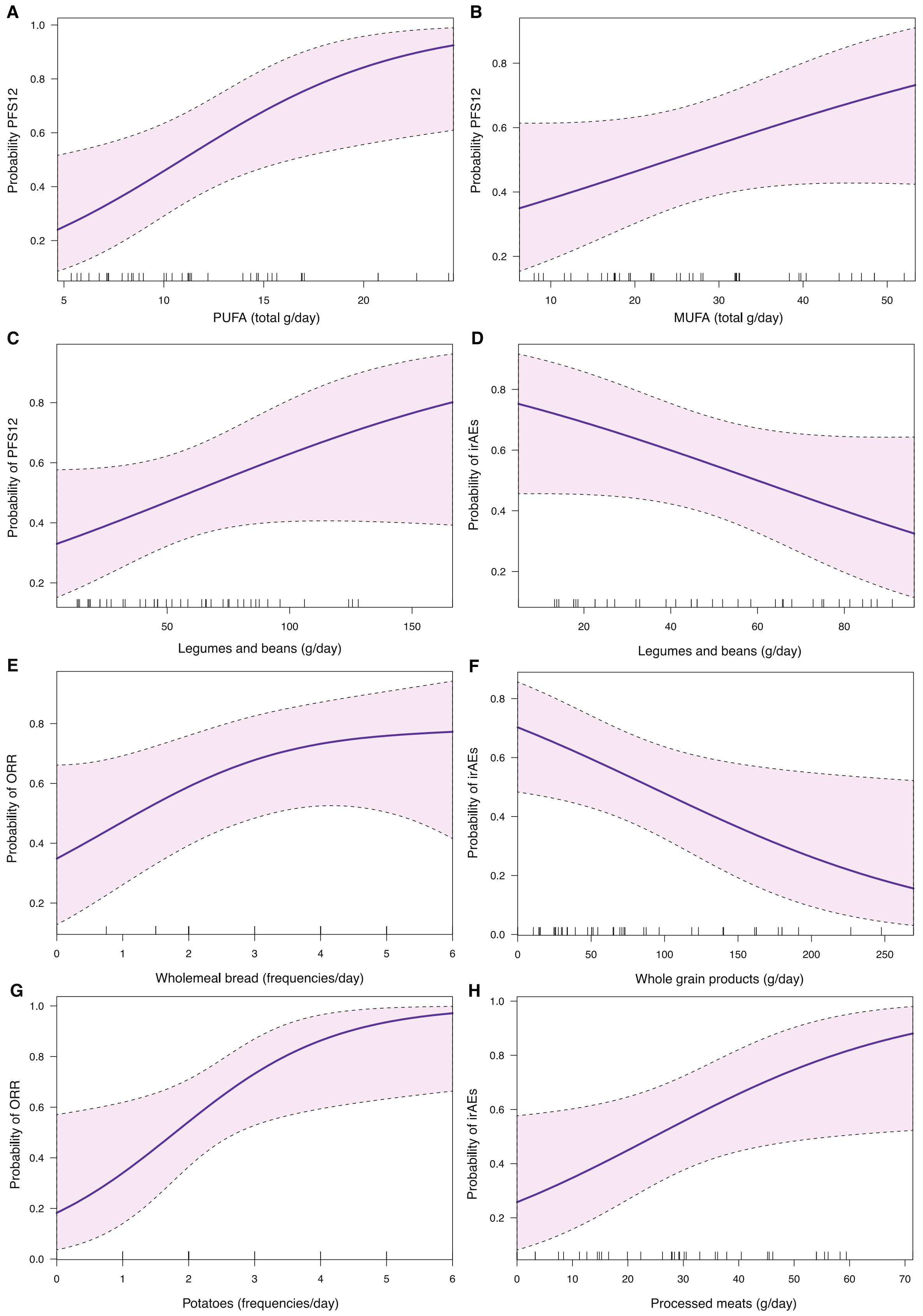


**eFigure 6. Relationships between treatment outcomes and specific nutrients and food groups.** Associations between treatment outcomes and specific dietary factors in PRIMM-UK (grams per day) and PRIMM-NL (frequencies per day) were not significant after multiple hypothesis testing correction (*P* <.05, *FDR* >.05, **eTables 8 and 9**). Abbreviations: PUFA, polyunsaturated fatty acids; MUFA, monounsaturated fatty acids; PFS-12, progression-free survival at 12 months; ORR, overall response rate; irAEs, immune-related adverse events; gr, grams.

© 2023 Bolte LA et al. *JAMA Oncology.*

eTable 1. Composition of food groups

| Food groups PRIMM-UK | **Single food items EPIC-Norfolk FFQ** | | **Food groups PRIMM-NL** | **Single food items DHD-FFQ** |
|---|---|---|---|---|
| Alcoholic beverages | red wine, white wine, beer, port, spirits | | Alcoholic beverages | alcoholic drinks on weekdays, alcoholic drinks on weekend days |
| Whole grain products | brown bread, wholemeal bread, brown rice, wholemeal pasta, porridge, high-fibre cereals | | Wholemeal bread | brown bread, wholemeal bread, multigrain bread |
| Refined grain products | white bread, naan bread/tortillas, white pasta, white rice, pizza, frosties, cereal, muesli, crispbread, crackers | | White bread | white bread |
| Sweets desserts and snacks | ice cream, dessert, puddings, chocolates, chocolate bars, sugar, jam, sweets, crisps, nuts salted, biscuit reduced fat, plain biscuit, chocolate biscuit, cake, cake, fruit pies, buns, sponge | | Biscuits and cakes | small cookies (biscuits, ginger cookie, shortbread, pretzel etc.), large cookies, cakes or pies (waffle, muffin, pie etc.) |
| Fats and oils | butter, hard margarine, polyunsaturated margarine, other margarine, low-fat spread, very low-fat spread, reduced fat butter, olive oil spread, cholesterol lowering spread | | Fats and oils | butter, margarine, cooking oil |
| Fish and fish products | oily fish, white fish, shellfish, fish fingers, fried fish | | Fish | fatty fish (like salmon, mackerel, herring), low-fat / white fish (tuna, cod, tilapia) |
| Poultry | chicken | | Meat | meat |
| Red and processed meat | beef, burger, pork, lamb, bacon, ham, corned beef, sausages, savoury pies, liver | | Cold cuts and spreads (fish, meat) | cold cuts of meat, fish on toast, meat-or fish-based spreads |
| Fruit | apples, pears, oranges, grapefruit, bananas, grapes, melons, peaches, berries, tinned fruit, dried fruit | | Fruit | fruit |
| Cheese | low-fat cheese, cottage cheese, cheese | | Cheese | high-fat cheese (48+, 40+, cream cheese), low-fat cheese (e.g. 30+, 20+) |
| Milk | milk | | Milk and milk-based drinks | milk, butter milk, chocolate milk, yoghurt drink, fruit-flavored milk |
| Fruit juice | fruit juice, smoothies | | Fruit juice | fruit juice |
| Sugar-sweetened beverages | fruit squash cordial, fizzy drinks, low-calorie fizzy drinks, chocolate drink, horlicks, cocoa, coffee whitener | | Savoury snacks and ready meals | potato chips, savoury snacks, cheese snack, pizza, ready meals, Asian-style meal |
| Potatoes | boiled potatoes, potato salad, roasted potatoes | | Potatoes | potatoes, boiled, baked or mashed |
| Sauces and condiments | salad cream, low-calorie salad cream, french, other dressing, sauces, ketchup, pickles, marmite | | Sauces | salt-containing sauce (like Maggi or ketchup) |
| Soup | meat soup, vegetable soup | | Soup | soup |

© 2023 Bolte LA et al. *JAMA Oncology*.

| Food groups PRIMM-UK | Single food items EPIC-Norfolk FFQ |
|---|---|
| Legumes and beans | tofu, green beans, peas, lentils, beansprouts, baked beans |
| Nuts and seeds unsalted | nuts unsalted, seeds |
| Eggs and egg dishes | eggs, quiche |
| Yoghurt | low-fat yogurt, full-fat yogurt |
| Tea | tea, fruit tea, green tea |
| Coffee | decaffeinated coffee, instant coffee, coffee |

eTable 1. Composition of food groups. Prior to performing a principal component analysis, single food items assessed by the EPIC-Norfolk FFQ and DHD-FFQ, respectively, were collapsed into standardized food groups using the national food composition databases [McCance and Widdowson composition of foods integrated dataset 2021, Public Health England (PHE); Dutch Food Composition Database (NEVO) 2021, National Institute for Public Health and the Environment in the Netherlands (RIVM)]. Abbreviations: PCA, Principal component analysis; FFQ, Food Frequency Questionnaire; DHD-FFQ, Dutch healthy diet FFQ; EPIC, European Prospective Investigation into Cancer and Nutrition.

© 2023 Bolte LA et al. *JAMA Oncology*.

eTable 2. Composition of the diet scores

| | EPIC-Norfolk FFQ | DHD-FFQ | aMED |
|---|---|---|---|
| *Positive food groups* | | | |
| Vegetables | carrots, sprouts, cabbage, marrow, leeks, cauliflower, parsnips, onions, garlic, green salad, spinach, mushrooms, peppers, tomatoes, sweetcorn, beetroot, coleslaw, avocado, broccoli, watercress, vegetable soup | vegetable boiled, vegetables stir-fried, vegetables raw | 1 point for intakes above median |
| Legumes and Nuts | tofu, green beans, peas, lentils, beansprouts, baked beans, nuts, seeds, peanut butter | n/A | 1 point for intakes above median |
| Fruit | apples, pears, oranges, grapefruit, bananas, grapes, melons, peaches, berries, tinned fruit, dried fruit | fruit | 1 point for intakes above median |
| Fish | oily fish, white fish, shellfish, fish fingers, fried fish | fatty fish (like salmon, mackerel, herring), low-fat / white fish (tuna, cod, tilapia), fish on toast | 1 point for intakes above median |
| Non-refined or high-fibre grains | brown bread, wholemeal bread, brown rice, wholemeal pasta, porridge, high-fibre cereal, muesli, crispbread | wholemeal bread, brown bread, multigrain bread | 1 point for intakes above median |
| MUFA/SFA ratio | ratio of monounsaturated to saturated fatty acids | n/A | 1 point for intakes above median |
| *Negative food groups* | | | |
| Red and processed meat | beef, burger, pork, lamb, bacon, ham, corned beef, sausages, savoury pies, liver | meats, cold cut meat | 1 point for intakes below median |
| Alcohol | red wine, white wine, liquor, spirits, port wine, beer -> converted into total alcohol in grams per day | alcoholic drinks on weekdays, alcoholic drinks on weekend days | 1 point for intakes > 5 and < 25 grams per day |

eTable 2. Composition of the alternate Mediterranean Diet score (aMED). Food items constituting the aMED food groups from EPIC-Norfolk FFQ and DHD-FFQ, respectively. We calculated an alternate Mediterranean aMED score, adapted from the original Mediterranean diet scale by Trichopoulou et al. (doi: 10.1056/NEJMoa025039; doi: 10.1136/bmj.311.7018.1457). One point was given for intakes above the median of each of the following components: fruit, vegetables, legumes and nuts, non-refined or high-fibre grains, fish and the ratio of monounsaturated to saturated fats. One point was given for intakes below the median of red and processed meat and for alcohol intakes between 5-25 grams per day. Food groups that were assessed in both cohorts were used to create a joint diet score. For comparability, we also calculated the 'full' diet score, i.e. making use of all food groups and nutrients assessed by the more extensive EPIC-Norfolk FFQ. Abbreviations: FFQ, Food Frequency Questionnaire; DHD-FFQ, Dutch healthy diet FFQ; EPIC, European Prospective Investigation into Cancer and Nutrition; MUFA/SFA, ratio of monounsaturated to saturated fatty acids; aMED, alternate Mediterranean diet score.

© 2023 Bolte LA et al. *JAMA Oncology*.

eTable 2. Composition of the diet scores

| | EPIC-Norfolk FFQ | DHD-FFQ | oPDI | hPDI | uPDI |
|---|---|---|---|---|---|
| *Plant-based foods* | | | | | |
| Whole grains | brown bread, wholemeal bread, brown rice, wholemeal pasta, porridge, high-fibre cereal, muesli, crispbread | wholemeal bread, brown bread, multigrain bread | positive scores | positive scores | reverse scores |
| Fruits | apples, pears, oranges, grapefruit, bananas, grapes, melons, peaches, berries, tinned fruit, dried fruit | fruit | positive scores | positive scores | reverse scores |
| Vegetables | carrots, cabbage, marrow, cauliflower, parsnips, leeks, onions, garlic, mushrooms, peppers, tomatoes, sweetcorn, beetroot, coleslaw, avocado, spinach, broccoli, green salad, watercress, sprouts, beansprouts, peas, green beans, vegetable soup, marmite, ketchup, pickles, sauces | cooked vegetables cooked, stir-fried vegetables, raw vegetables | positive scores | positive scores | reverse scores |
| Nuts and legumes | nuts, seeds, peanut butter, tofu, lentils, baked beans | n/A | positive scores | positive scores | reverse scores |
| Vegetable oils | french dressing, olive oil spread, other dressing | (cooking oil) | positive scores | positive scores | reverse scores |
| Tea and coffee | decaffeinated coffee, instant coffee, coffee, tea, fruit tea, green tea | n/A | positive scores | positive scores | reverse scores |
| *Less healthy plant-based foods* | | | | | |
| Fruit juices | fruit juice, fruit concentrate | fruit juice | positive scores | reverse scores | positive scores |
| Refined grains | muesli, naan bread/tortillas, white bread, white pasta, white rice, crispbread, cornflakes, frosties, crackers | white bread | positive scores | reverse scores | positive scores |
| Potatoes | boiled potatoes, roasted potatoes, potato salad, crisps | potatoes, boiled, baked or mashed, crisps | positive scores | reverse scores | positive scores |
| Sugar sweetened beverages | soft drinks, fruit squash, low-calorie fizzy drinks, cocoa, coffee whitener, horlicks, hot chocolate | n/A | positive scores | reverse scores | positive scores |
| Sweets and Desserts | biscuits reduced fat, cereal bars, sugar topped cereals, chocolate bars, chocolate biscuits, chocolate, cake, sponge, fruit pies, plain biscuit, buns, sugar, sweets, jam | small cookies (biscuits, ginger cookie, shortbread, pretzel etc.), large cookies, cakes or pies (waffle, muffin, pie etc.) | positive scores | reverse scores | positive scores |

© 2023 Bolte LA et al. *JAMA Oncology*.

| | EPIC-Norfolk FFQ | DHD-FFQ | oPDI | hPDI | uPDI |
|---|---|---|---|---|---|
| *Animal Food Groups* | | | | | |
| Animal fat | butter, butter reduced fat | high-fat cheese on bread, high-fat cheese snack, whole milk | reverse scores | reverse scores | reverse scores |
| Dairy | cheese reduced fat, cottage cheese, low-fat yogurt, cheese, dairy-based desserts, milk puddings, double cream, single cream, full fat yogurt, ice cream | cheese on bread, cheese snacks, milk, butter milk, chocolate milk, yoghurt drink, fruit-flavored milk | reverse scores | reverse scores | reverse scores |
| Egg | boiled eggs | n/A | reverse scores | reverse scores | reverse scores |
| Fish or Seafood | oily fish, white fish, shellfish, fish fingers, fried fish | fatty fish (like salmon, mackerel, herring), low-fat / white fish (tuna, cod, tilapia), fish on toast | reverse scores | reverse scores | reverse scores |
| Meat | poultry, meat soup, beef, burger, pork, lamb, bacon, ham, corned beef, sausages, savoury pies, liver, lasagna | meat | reverse scores | reverse scores | reverse scores |
| Miscellaneous animal-based foods | salad cream, low-calorie salad cream, pizza, quiche, savoury/meat pies, mayonnaise | cold cut meat, meat-and fish spreads | reverse scores | reverse scores | reverse scores |

**eTable 2. Composition of the Plant-based diet indices (PDI).** Food items constituting the food groups from the EPIC-Norfolk FFQ and DHD-FFQ required for calculating the plant-based diet indices. Food groups were created within the larger categories of healthy plant foods, less healthy plant foods and animal foods, using the method by Satija et al. (doi:10.1016/j.jacc.2017.05.047). Food groups were ranked into quintiles, and given positive or reverse scores. With positive scores, participants above the highest quintile of a food group received a score of 5, following on through to participants below the lowest quintile who received a score of 1. With reverse scores, this pattern of scoring was inversed. For creating the original PDI, plant food groups were given positive scores, while animal food groups were given reverse scores. For creating hPDI, positive scores were given to healthy plant food groups, and reverse scores to less healthy plant food groups and animal food groups. Finally, for uPDI, positive scores were given to less healthy plant food groups, and reverse scores to healthy plant food groups and animal food groups. The food group scores were summed to obtain the indices. Higher intake of all indices reflected lower animal food intake e.g. 5–6 vs. 3 servings/day comparing extreme PDI deciles. Food groups that were assessed in both cohorts were used to create a joint diet score. For comparability, we also calculated the 'full' diet score, i.e. making use of all food groups and nutrients assessed by the more extensive EPIC-Norfolk FFQ. Abbreviations: FFQ, Food Frequency Questionnaire; DHD-FFQ, Dutch healthy diet FFQ; EPIC, European Prospective Investigation into Cancer and Nutrition; oPDI, Overall Plant-based Diet Index; hPDI, Healthy Plant-based Diet Index; uPDI, Unhealthy Plant-based Diet Index.

© 2023 Bolte LA et al. *JAMA Oncology*.

## eTable 3. Backward selection for variable importance

**Per country: PRIMM-UK**

| | PC1 | PC2 | PC3 | PC4 | PC5 | Deviance explained by model including all PCs |
|---|---|---|---|---|---|---|
| **PFS12** | 56.8 | 39.8 | 0.0 | 0.0 | 0.0 | 5.9 |
| **ORR** | 0.0 | 100.0 | 0.0 | 0.0 | 0.0 | 2.2 |
| **irAEs** | 0.0 | 49.9 | 0.0 | 0.0 | 0.0 | 23.5 |

**Per country: PRIMM-NL**

| | PC1 | PC2 | PC3 | PC4 | PC5 | Deviance explained by model including all PCs |
|---|---|---|---|---|---|---|
| **PFS12** | 0.0 | 61.0 | 0.0 | 1.1 | 0.4 | 58.3 |
| **ORR** | 12.2 | 51.1 | 0.0 | 0.0 | 0.0 | 50.8 |
| **irAEs** | 0.0 | 26.8 | 23.3 | 57.0 | 0.0 | 18.7 |

**Joint PRIMM-UK & PRIMM-NL**

| | aMED | oPDI | hPDI | uPDI | Deviance explained by model including all diet scores |
|---|---|---|---|---|---|
| **PFS12** | 54.1 | 0.0 | 0.0 | 0.0 | 13.7 |
| **ORR** | 51.2 | 0.0 | 0.0 | 0.0 | 8.5 |
| **irAEs** | 24.0 | 0.0 | 0.0 | 12.4 | 15.4 |

**Per country: PRIMM-UK**

| | aMED | oPDI | hPDI | uPDI | Deviance explained by model including all diet scores |
|---|---|---|---|---|---|
| **PFS12** | 88.3 | 0.0 | 17.5 | 0.0 | 8.1 |
| **ORR** | 100.0 | 0.0 | 0.0 | 0.0 | 1.1 |
| **irAEs** | 56.5 | 0.0 | 0.0 | 0.0 | 11.8 |

**Per country: PRIMM-NL**

| | aMED | oPDI | hPDI | uPDI | Deviance explained by model including all diet scores |
|---|---|---|---|---|---|
| **PFS12** | 26.1 | 0.0 | 26.8 | 0.0 | 41.6 |
| **ORR** | 73.3 | 0.0 | 39.1 | 0.0 | 74.0 |
| **irAEs** | 37.1 | 0.0 | 0.0 | 7.7 | 8.0 |

**eTable 3. Backward selection for variable importance.** To test which dietary pattern had the largest influence on response and irAEs, we removed each diet score or PC from each model one at a time, keeping all other variables intact. Removing a dietary pattern with a low explanatory power will do little to the deviance explained, while removing a dietary pattern with a large explanatory power will substantially reduce the deviance explained by the focal model. Tables show the loss in (%) deviance from full model as we successively removed each predictor variable from the model keeping the model otherwise intact. Abbreviations: PFS12, Progression-free survival at 12 months; ORR, Overall response rate; irAEs, immune-related adverse events; aMED, alternate Mediterranean diet score; oPDI, Overall Plant-based Diet Index; hPDI, Healthy Plant-based Diet Index; uPDI, Unhealthy Plant-based Diet Index.

© 2023 Bolte LA et al. *JAMA Oncology*.

eTable 4. Descriptive statistics of the PRIMM-cohorts

| | **Total PRIMM cohort (n=91)** | **PRIMM-NL (n=44)** | **PRIMM-UK (n=47)** | **Non-zeros PRIMM-NL** | **Non-zeros PRIMM-UK** | ***P-value*** | ***FDR*** |
|---|---|---|---|---|---|---|---|
| Age (years) at stage IV diagnosis, mean (SD) | 62.93 (15.18) | 59.43 (12.74) | 66.21 (16.63) | 44 | 47 | 0.020 | 0.630 |
| BMI (kg/m2), mean (SD) | 28.31 (5.46) | 27.51 (5.55) | 29.06 (5.32) | 44 | 47 | 0.189 | 1 |
| BMI categories, n (%) | | | | 44 | 47 | 0.635 | 1 |
| underweight (BMI <20) | 5 (5.49) | 4 (9.09) | 1 (2.13) | | | n/A | n/A |
| normal weight (BMI 20-25) | 23 (25.27) | 12 (27.27) | 11 (23.40) | | | n/A | n/A |
| overweight (BMI 25-30) | 34 (37.36) | 15 (34.09) | 19 (40.43) | | | n/A | n/A |
| obesity (BMI >30) | 27 (29.27) | 12 (27.27) | 15 (31.91) | | | n/A | n/A |
| morbid obesity (BMI >35) | 2 (2.20) | 1 (2.27) | 1 (2.13) | | | n/A | n/A |
| Gender, n (%) | | | | 44 | 47 | 0.123 | 1 |
| male | 54 (59.34) | 22 (50.00) | 32 (68.09) | | | n/A | n/A |
| female | 37 (40.66) | 22 (50.00) | 15 (31.91) | | | n/A | n/A |
| Outcomes following ICB, n (%) | | | | 44 | 47 | | |
| PFS-12 | 43 (47.25) | 20 (45.45) | 23 (48.94) | | | 0.930 | 1 |
| ORR | 53 (58.24) | 26 (59.09) | 27 (57.45) | | | 1 | 1 |
| irAEs grade ≥ 2 | 46 (50.55) | 21 (47.73) | 25 (53.19) | | | 0.756 | 1 |
| Colitis grade ≥ 2 | 12 (13.19) | 3 (6.82) | 9 (19.15) | | | 0.153 | 1 |
| Maximum grade of severity of irAEs | | | | 44 | 47 | 0.264 | 1 |
| no irAEs | 26 (28.57) | 14 (31.82) | 12 (25.53) | | | n/A | n/A |
| grade 1 | 19 (20.88) | 9 (20.45) | 10 (21.28) | | | n/A | n/A |
| grade 2 | 15 (16.48) | 10 (22.73) | 5 (10.64) | | | n/A | n/A |
| grade 3 | 28 (30.77) | 10 (22.73) | 18 (38.30) | | | n/A | n/A |
| grade 4 | 3 (3.30) | 1 (2.27) | 2 (4.26) | | | n/A | n/A |
| Number of different organs affected by irAEs | | | | 44 | 47 | 0.204 | 1 |
| no irAEs | 26 (28.57) | 14 (31.82) | 12 (25.53) | | | n/A | n/A |
| 1 | 36 (39.56) | 19 (43.18) | 17 (36.17) | | | n/A | n/A |
| 2 | 19 (20.88) | 8 (18.18) | 11 (23.40) | | | n/A | n/A |
| 3 | 8 (8.79) | 2 (4.55) | 6 (12.77) | | | n/A | n/A |
| 4 | 2 (2.20) | 1 (2.27) | 1 (2.13) | | | n/A | n/A |

© 2023 Bolte LA et al. *JAMA Oncology*.

| | **Total PRIMM cohort (n=91)** | **PRIMM-NL (n=44)** | **PRIMM-UK (n=47)** | **Non-zeros PRIMM-NL** | **Non-zeros PRIMM-UK** | ***P-value*** | ***FDR*** |
|---|---|---|---|---|---|---|---|
| Metastatic stage, n (%) | | | | 44 | 47 | **0.014** | 0.435 |
| Stage 3, unresectable | 5 (5.49) | 1 (2.27) | 4 (8.51) | | | n/A | n/A |
| M1a | 17 (18.68) | 6 (13.64) | 11 (23.40) | | | n/A | n/A |
| M1b | 19 (20.88) | 8 (18.18) | 11 (23.40) | | | n/A | n/A |
| M1c | (29 (31.87) | 12 (27.27) | 17 (36.17) | | | n/A | n/A |
| M1d | 21 (23.08) | 17 (38.64) | 4 (8.51) | | | n/A | n/A |
| BRAF mutant, n (%) | 37 (40.66) | 23 (52.27) | 14 (29.79) | | | 0.049 | 1 |
| ECOG Performance score ≥1, n (%) | 49 (53.85) | 16 (36.36) | 33 (70.21) | | | 0.002 | 0.079 |
| ICB used, n (%) | | | | 44 | 47 | 0.043 | 1 |
| Single agent PD-1/PDL-1 inhibition | 56 (61.54) | 32 (72.73) | 24 (51.06) | | | n/A | n/A |
| Single agent CTLA-4 inhibition | 1 (1.10) | 1 (2.27) | 0 (0.00) | | | n/A | n/A |
| Ipilimumab-nivolumab combination | 34 (37.36) | 11 (25.00) | 23 (48.94) | | | n/A | n/A |
| Previous BRAF or MEK inhibition, n (%) | 26 (28.57) | 17 (38.64) | 9 (19.15) | | | 0.068 | 1 |
| Antibiotic use at baseline, n (%) | 18 (20.45) | 10 (23.26) | 8 (17.78) | | | 0.71 | 1 |
| PPI use at baseline, n (%) | 31 (34.07) | 19 (43.18) | 12 (25.53) | | | 0.12 | 1 |
| Diet scores, mean (SD) | | | | 44 | 47 | | |
| aMED | 2.8 (1.28) | 3.07 (1.25) | 2.55 (1.28) | | | 0.083 | 1 |
| OriginalPDI | 32.44 (4.74) | 30.52 (4.29) | 34.23 (4.45) | | | 1.22E-04 | **0.004** |
| hPDI | 34.21 (6.76) | 32.84 (5.81) | 35.49 (7.37) | | | 0.13 | 1 |
| uPDI | 33.05 (5.29) | 31.70 (4.56) | 34.32 (5.65) | | | 0.021 | 0.677 |
| Distribution of the aMED score, n (%) | | | | 44 | 47 | | |
| 0 | 4 (4.40) | 0 (0.00) | 4 (8.51) | | | | |
| 1 | 9 (9.89) | 5 (11.36) | 4 (8.51) | | | | |
| 2 | 24 (26.37) | 10 (22.73) | 14 (29.79) | | | | |
| 3 | 28 (30.77) | 13 (29.55) | 15 (31.91) | | | | |
| 4 | 16 (17.58) | 9 (20.45) | 7 (14.89) | | | | |
| 5 | 10 (11.00) | 7 (15.91) | 3 (6.38) | | | | |
| Food groups score components, mean (SD) | | | | 44 | 47 | | |
| Vegetables PDI | 2.62 (1.32) | 2.23 (1.01) | 2.98 (1.48) | | | 0.015 | 0.492 |
| Whole grain PDI | 2.85 (1.51) | 2.68 (1.57) | 3.00 (1.46) | | | 0.290 | 1 |
| Fruit PDI | 2.51 (1.23) | 2.00 (0.65) | 2.98 (1.45) | | | 0.001 | **0.034** |
| Fruit juices PDI | 2.04 (1.16) | 2.09 (1.07) | 2.00 (1.25) | | | 0.529 | 1 |

© 2023 Bolte LA et al. *JAMA Oncology*.

| | | | | | | | |
|---|---|---|---|---|---|---|---|
| Refined grains PDI | 2.12 (1.39) | 1.20 (0.41) | 2.98 (1.44) | | | 5.47E-10 | **1.75E-08** |
| | **Total PRIMM cohort (n=91)** | **PRIMM-NL (n=44)** | **PRIMM-UK (n=47)** | **Non-zeros PRIMM-NL** | **Non-zeros PRIMM-UK** | ***P-value*** | ***FDR*** |
| Potatoes PDI | 2.84 (1.47) | 2.77 (1.45) | 2.89 (1.51) | | | 0.742 | 1 |
| Sweets, desserts PDI | 2.89 (1.44) | 2.77 (1.43) | 3.00 (1.46) | | | 0.467 | 1 |
| Animal fats PDI | 2.84 (1.43) | 2.80 (1.41) | 2.87 (1.47) | | | 0.783 | 1 |
| Dairy PDI | 2.81 (1.44) | 2.64 (1.38) | 2.98 (1.48) | | | 0.259 | 1 |
| Fish and seafood PDI | 2.9 (1.48) | 2.98 (1.42) | 2.83 (1.54) | | | 0.621 | 1 |
| Meat PDI | 2.18 (1.39) | 1.36 (0.65) | 2.94 (1.47) | | | 4.43E-08 | **1.42E-06** |
| Miscellaneous animal foods PDI | 1.73 (1.07) | 1.45 (0.70) | 1.98 (1.28) | | | 0.040 | 1 |
| Nutritional status, mean (SD) | | | | | | | |
| Serum albumin | 41.89 (3.92) | 42.60 (3.06) | 41.43 (4.36) | 30 | 46 | 0.222 | 1 |
| Total caloric intake | n/A | n/A | 1726.85 (2057.94) | 0 | 47 | n/A | n/A |
| EI/BMR ratio | n/A | n/A | 1.033 (1.29) | 0 | 47 | n/A | n/A |
| Protein en-% | n/A | n/A | 17.13 (18.27) | 0 | 47 | n/A | n/A |
| Protein grams/kg bodyweight | n/A | n/A | 0.89 (1.08) | 0 | 47 | n/A | n/A |
| Carbohydrates en-% | n/A | n/A | 44.69 (44.93) | 0 | 47 | n/A | n/A |
| Fat en-% | n/A | n/A | 37.72 (37.10) | 0 | 47 | n/A | n/A |

eTable 4. Descriptive statistics of the PRIMM cohorts. Baseline characteristics are presented as mean and standard deviation (SD) for continuous variables and as counts and percentages for categorical variables. $\chi^2$ tests for categorical variables and Mann-Whitney U test (MWU) for continuous data were performed to calculate differences between cohorts. P-values written in bold indicate nominally significant differences between PRIMM-UK and PRIMM-NL ($P < .05$). FDR values written in bold indicate statistical significance under a false discovery rate (FDR) of 5%. Non-zeros indicate availability of phenotypes per cohort. Macronutrients were only available for the PRIMM-UK dataset. Abbreviations: BMI, body-mass index; aMED, alternate Mediterranean diet score; oPDI, Overall Plant-based Diet Index; hPDI, Healthy Plant-based Diet Index; uPDI, Unhealthy Plant-based Diet Index; ICB, immune checkpoint blockade; ECOG, Eastern Cooperative Oncology Group; BRAF, v-raf murine sarcoma viral oncogene homolog B1, MEK, mitogen-activated protein kinase; PFS12, progression-free survival at 12 months; ORR, overall response rate; irAE, immune related adverse event; en-%, energy-%, SD, standard deviation.

© 2023 Bolte LA et al. *JAMA Oncology*.

eTable 4. Descriptive statistics of Responders and Non-Responders

| | Responders (n=53) | Non-Responders (n=38) | Non-zeros Responders | Non-zeros Non-Responders | ***P-value*** | ***FDR*** |
|---|---|---|---|---|---|---|
| Age (years) at stage IV diagnosis, mean (SD) | 62.89 (13.86) | 63 (17.04) | 53 | 38 | 0.618 | 1 |
| BMI (kg/m2), mean (SD) | 28.16 (5.33) | 28.52 (5.69) | 53 | 38 | 0.901 | 1 |
| BMI categories, n (%) | | | 53 | 38 | | |
| underweight (BMI <20) | 4 (7.55) | 1 (2.63) | | | n/A | n/A |
| normal weight (BMI 20-25) | 14 (26.42) | 9 (23.68) | | | n/A | n/A |
| overweight (BMI 25-30) | 17 (32.08) | 17 (44.74) | | | n/A | n/A |
| obesity (BMI >30) | 18 (33.96) | 9 (23.68) | | | n/A | n/A |
| morbid obesity (BMI >35) | 0 (0) | 2 (5.26) | | | n/A | n/A |
| Gender, n (%) | | | 53 | 38 | 0.032 | 0.579 |
| Male | 26 (49.06) | 28 (73.68) | | | n/A | n/A |
| Female | 27 (50.94) | 10 (26.32) | | | n/A | n/A |
| Outcomes following ICB, n (%) | | | | | | |
| irAEs grade ≥ 2 | 27 (50.94) | 19 (50.00) | 53 | 38 | 1 | 1 |
| Colitis grade ≥ 2 | 5 (9.43) | 7 (18.42) | 53 | 38 | 0.350 | 1 |
| Maximum grade of severity of irAEs | | | 53 | 38 | 0.080 | 1 |
| no irAEs | 9 (16.98) | 17 (44.74) | | | n/A | n/A |
| grade 1 | 17 (32.08) | 2 (5.26) | | | n/A | n/A |
| grade 2 | 9 (16.98) | 6 (15.79) | | | n/A | n/A |
| grade 3 | 17 (32.08) | 11 (28.95) | | | n/A | n/A |
| grade 4 | 1 (1.89) | 2 (5.26) | | | n/A | n/A |
| Number of different organs affected by irAEs | | | 53 | 38 | 0.869 | 1 |
| none | 9 (16.98) | 17 (44.74) | | | n/A | n/A |
| 1 | 25 (47.17) | 11 (28.95) | | | n/A | n/A |
| 2 | 12 (22.64) | 7 (18.42) | | | n/A | n/A |
| 3 | 5 (9.43) | 3 (7.89) | | | n/A | n/A |
| 4 | 2 (3.77) | 0 (0.00) | | | n/A | n/A |

© 2023 Bolte LA et al. *JAMA Oncology*.

| | Responders (n=53) | Non-Responders (n=38) | Non-zeros Responders | Non-zeros Non-Responders | *P-value* | *FDR* |
|---|---|---|---|---|---|---|
| Metastatic stage, n (%) | | | | | 0.233 | 1 |
| Stage 3, unresectable | 3 (5.66) | 2 (5.26) | 53 | 38 | n/A | n/A |
| M1a | 13 (24.53) | 4 (10.53) | 53 | 38 | n/A | n/A |
| M1b | 13 (24.53) | 6 (15.79) | 53 | 38 | n/A | n/A |
| M1c | 13 (24.53) | 16 (42.11) | 53 | 38 | n/A | n/A |
| M1d | 11 (20.75) | 10 (26.32) | 53 | 38 | n/A | n/A |
| BRAF mutant, n (%) | 19 (35.85) | 18 (47.37) | 53 | 38 | 0.375 | 1 |
| ECOG Performance score ≥1, n (%) | 27 (50.94) | 22 (57.89) | 53 | 38 | 0.658 | 1 |
| ICB used, n (%) | | | | | 0.687 | 1 |
| Single agent PD-1/PDL-1 inhibition | 32 (60.38) | 24 (63.16) | 53 | 38 | n/A | n/A |
| Single agent CTLA-4 inhibition | 1 (1.89) | 0 (0.00) | 53 | 38 | n/A | n/A |
| Ipilimumab-nivolumab combination | 20 (37.74) | 14 (36.84) | 53 | 38 | n/A | n/A |
| Previous BRAF or MEK inhibition, n (%) | 9 (16.98) | 17 (44.74) | 53 | 38 | 0.008 | 0.143 |
| Antibiotic use at baseline, n (%) | 8 (15.69) | 10 (27.03) | 51 | 37 | 0.301 | 1 |
| PPI use at baseline, n (%) | 17 (32.08) | 14 (36.84) | 53 | 38 | 0.803 | 1 |
| Nutritional status, mean (SD) | | | | | | |
| Serum albumin | 42.47 (4.20) | 41.15 (3.45) | 43 | 33 | 0.108 | 1 |
| Total caloric intake | 1883.07 (814.62) | 2294.02 (1815.21) | 27 | 20 | 0.940 | 1 |
| EI/BMR ratio | 1.19 (0.57) | 1.42 (1.28) | 27 | 20 | 0.940 | 1 |
| Protein en-% | 17.39 (2.92) | 19.45 (5.73) | 27 | 20 | 0.049 | 0.980 |
| Protein grams/kg bodyweight | 0.99 (0.50) | 1.2 (0.83) | 27 | 20 | 0.628 | 1 |
| Carbohydrates en-% | 44.65 (6.40) | 45.31 (9.4) | 27 | 20 | 0.471 | 1 |
| Fat en-% | 38.02 (5.58) | 35.85 (7.71) | 27 | 20 | 0.186 | 1 |

eTable 4. Descriptive Statistics of Responders and Non-Responders. Patients were classified as responders using the Response Evaluation Criteria in Solid Tumors (RECIST) v1.1 criteria. Baseline characteristics are presented as mean and standard deviation (SD) for continuous variables and as counts and percentages for categorical variables. χ2 tests for categorical variables and Mann-Whitney U test (MWU) for continuous data were performed to calculate differences between cohorts. P-values written in bold indicate nominally significant differences between Responders and Non-Responders (P < .05). FDR values written in bold indicate statistical significance under a false discovery rate (FDR) of 5%. Non-zeros indicate availability of phenotypes per subgroup. Macronutrients were only available for the PRIMM-UK dataset. Abbreviations: BMI, body-mass index; aMED, alternate Mediterranean diet score; oPDI, Overall Plant-based Diet Index; hPDI, Healthy Plant-based Diet Index; uPDI, Unhealthy Plant-based Diet Index. ICB, immune checkpoint

© 2023 Bolte LA et al. *JAMA Oncology*.

| |
|---|
| blockade; ECOG, Eastern Cooperative Oncology Group; BRAF, v-raf murine sarcoma viral oncogene homolog B1, MEK, mitogen-activated protein kinase; PFS12, progression-free survival at 12 months; ORR, overall response rate; en-%, energy-%, SD, standard deviation. |

© 2023 Bolte LA et al. *JAMA Oncology*.

eTable 5. Results of the generalized additive models using diet scores

| Joint PRIMM-UK & PRIMM-NL | | | | | | **Per country: PRIMM-UK** | | | | | **Per country: PRIMM-NL** | | | | |
|---|---|---|---|---|---|---|---|---|---|---|---|---|---|---|---|
| PFS12 | | | | | | **PFS12** | | | | | **PFS12** | | | | |
| term | **edf** | **ref.df** | **Chi.sq** | **p.value** | **FDR** | **edf** | **ref.df** | **Chi.sq** | **p.value** | **FDR** | **edf** | **ref.df** | **Chi.sq** | **p.value** | **FDR** |
| s(aMED) | 1.540 | 4 | 7.540 | 0.007 | 0.021 | 1.290 | 4 | 3.220 | 0.078 | 0.143 | 0.874 | 4 | 9.180 | 0.013 | 0.020 |
| s(oPDI) | 5.39E-06 | 4 | 2.00E-06 | 0.632 | 0.948 | 8.23E-06 | 4 | 2.78E-06 | 0.685 | 0.729 | 5.37E-06 | 4 | 1.15E-06 | 0.760 | 0.760 |
| s(hPDI) | 1.83E-05 | 4 | 9.67E-06 | 0.542 | 0.992 | 0.353 | 4 | 0.531 | 0.219 | 0.657 | 1.670 | 4 | 5.900 | 0.060 | 0.090 |
| s(uPDI) | 8.56E-06 | 4 | 5.15E-07 | 0.949 | 0.949 | 8.12E-06 | 4 | 6.02E-07 | 0.932 | 0.999 | 5.62E-06 | 4 | 9.80E-07 | 0.815 | 0.815 |
| s(bmi) | 1.100 | 4 | 2.590 | 0.094 | 0.282 | 3.69E-05 | 4 | 3.29E-05 | 0.380 | 0.570 | 1.02E-05 | 4 | 3.83E-06 | 0.610 | 0.679 |
| s(age) | 5.51E-06 | 4 | 1.07E-06 | 0.796 | 0.807 | 1.22E-06 | 4 | 3.89E-07 | 0.595 | 0.806 | 1.250 | 4 | 2.610 | 0.152 | 0.449 |
| s(sex) | 0.850 | 1 | 5.270 | 0.012 | 0.036 | 1.29E-05 | 1 | 1.09E-05 | 0.354 | 0.541 | 0.909 | 1 | 7.660 | 0.002 | 0.003 |
| s(centre) | 0.053 | 1 | 0.057 | 0.301 | 0.824 | | | | | | | | | | |
| | | | | | | | | | | | | | | | |
| ORR | | | | | | **ORR** | | | | | **ORR** | | | | |
| term | **edf** | **ref.df** | **Chi.sq** | **p.value** | **FDR** | **edf** | **ref.df** | **Chi.sq** | **p.value** | **FDR** | **edf** | **ref.df** | **Chi.sq** | **p.value** | **FDR** |
| s(aMED) | 0.826 | 4 | 4.460 | 0.021 | 0.032 | 0.296 | 4 | 4.109e-01 | 0.238 | 0.238 | 3.376 | 4 | 2.576+01 | 0.003 | 0.009 |
| s(oPDI) | 2.09E-05 | 4 | 1.33E-05 | 0.483 | 0.948 | 9.049e-06 | 4 | 3.694e-06 | 0.636 | 0.729 | 5.37E-06 | 4 | 1.315E-06 | 0.707 | 0.760 |
| s(hPDI) | 1.08E-05 | 4 | 2.30E-06 | 0.788 | 0.992 | 4.683e-06 | 4 | 3.886e-07 | 0.918 | 0.918 | 1.670 | 4 | 3.496E+01 | 0.008 | 0.024 |
| s(uPDI) | 1.44E-05 | 4 | 3.48E-06 | 0.765 | 0.949 | 5.585e-06 | 4 | 5.585e-06 | 0.999 | 0.999 | 5.62E-06 | 4 | 1.344E-06 | 0.628 | 0.815 |
| s(bmi) | 5.77E-06 | 4 | 9.01E-07 | 0.843 | 0.843 | 7.380e-06 | 4 | 2.785e-06 | 0.658 | 0.658 | 1.02E-05 | 4 | 6.208 | 0.033 | 0.099 |
| s(age) | 2.57E-06 | 4 | 4.40E-07 | 0.807 | 0.807 | 5.155e-07 | 4 | 6.661e-08 | 0.806 | 0.806 | 1.250 | 4 | 2.299E-05 | 0.431 | 0.449 |
| s(sex) | 0.815 | 1 | 4.170 | 0.024 | 0.036 | 5.434e-06 | 1 | 3.571e-06 | 0.415 | 0.541 | 0.909 | 1 | 8.339 | 0.001 | 0.003 |
| s(centre) | 8.13E-06 | 1 | 1.99E-06 | 0.620 | 0.824 | | | | | | | | | | |
| | | | | | | | | | | | | | | | |
| irAEs | | | | | | **irAEs** | | | | | **irAEs** | | | | |
| term | **edf** | **ref.df** | **Chi.sq** | **p.value** | **FDR** | **edf** | **ref.df** | **Chi.sq** | **p.value** | **FDR** | **edf** | **ref.df** | **Chi.sq** | **p.value** | **FDR** |
| s(aMED) | 1.140 | 4 | 2.530 | 0.091 | 0.091 | 1.360 | 4 | 3.130 | 0.095 | 0.143 | 0.574 | 4 | 1.420 | 0.135 | 0.135 |
| s(oPDI) | 7.37E-06 | 4 | 1.98E-07 | 0.984 | 0.948 | 9.20E-06 | 4 | 2.61E-06 | 0.729 | 0.729 | 1.96E-06 | 4 | 5.38E-07 | 0.726 | 0.760 |
| s(hPDI) | 6.42E-06 | 4 | 1.07E-07 | 0.992 | 0.992 | 6.36E-06 | 4 | 6.10E-07 | 0.902 | 0.918 | 3.17E-06 | 4 | 8.33E-08 | 0.984 | 0.984 |
| s(uPDI) | 0.726 | 4 | 1.070 | 0.201 | 0.603 | 8.63E-06 | 4 | 4.62E-06 | 0.525 | 0.999 | 0.192 | 4 | 0.215 | 0.310 | 0.815 |
| s(bmi) | 8.95E-06 | 4 | 2.01E-06 | 0.769 | 0.843 | 0.187 | 4 | 0.223 | 0.273 | 0.570 | 4.58E-06 | 4 | 1.56E-06 | 0.679 | 0.679 |
| s(age) | 0.715 | 4 | 2.750 | 0.072 | 0.216 | 0.660 | 4 | 1.710 | 0.106 | 0.318 | 1.83E-05 | 4 | 1.37E-05 | 0.449 | 0.449 |
| s(sex) | 0.762 | 1 | 2.980 | 0.046 | 0.046 | 3.77E-06 | 1 | 1.38E-06 | 0.541 | 0.541 | 0.730 | 1 | 2.510 | 0.061 | 0.061 |
| s(centre) | 3.05E-06 | 1 | 1.47E-07 | 0.824 | 0.824 | | | | | | | | | | |

eTable5. Results of the generalized additive models using diet scores. Logistic generalized additive models (GAMs) were used to determine whether a higher adherence to a particular diet (as assessed by different diet scores) is associated with a higher probability of response or irAEs. Using the joint dataset, we modelled each outcome variable (ORR, PFS-12, irAEs) and all four diet scores as independent variables adjusting for age, sex, BMI and cohort. Next, we repeated the analysis separately per cohort. Abbreviations: PFS12,

© 2023 Bolte LA et al. *JAMA Oncology*.

| |
|---|
| Progression-free survival at 12 months; ORR, Overall response rate; irAEs, immune-related adverse events; aMED, alternate Mediterranean diet score; oPDI, Overall Plant-based Diet Index; hPDI, Healthy Plant-based Diet Index; uPDI, Unhealthy Plant-based Diet Index. |

© 2023 Bolte LA et al. *JAMA Oncology*.

eTable 6. Loadings of food groups per dietary pattern identified by PCA and their correlations in PRIMM-UK

| Food groups | **PC1** | **PC2** | **PC3** | **PC4** | **PC5** |
|---|---|---|---|---|---|
| Alcoholic beverages | 0.252 | -0.151 | 0.070 | -0.054 | 0.190 |
| Eggs and egg dishes | 0.181 | -0.239 | -0.148 | -0.135 | -0.264 |
| Whole grain products | 0.121 | -0.031 | 0.355 | 0.300 | -0.284 |
| Refined grain products | 0.186 | 0.224 | 0.181 | -0.394 | 0.017 |
| Sweets, desserts and snacks | 0.324 | 0.163 | -0.222 | 0.165 | -0.214 |
| Fats and oils | 0.379 | 0.122 | -0.050 | 0.058 | 0.115 |
| Fish and fish products | 0.177 | -0.235 | -0.173 | -0.123 | -0.319 |
| Poultry | -0.084 | 0.091 | 0.137 | 0.285 | -0.237 |
| Red and processed meat | 0.385 | 0.220 | -0.024 | -0.094 | -0.104 |
| Fruit | -0.051 | -0.402 | 0.047 | 0.033 | -0.004 |
| Yoghurt | 0.064 | -0.033 | 0.298 | -0.416 | 0.030 |
| Cheese | 0.106 | -0.032 | 0.168 | 0.284 | 0.311 |
| Milk | 0.068 | 0.028 | -0.346 | 0.042 | -0.373 |
| Fruit juice | 0.179 | 0.091 | 0.410 | -0.275 | -0.215 |
| Sugar-sweetened beverages | 0.064 | 0.346 | -0.195 | -0.045 | 0.067 |
| Tea | 0.193 | -0.102 | 0.207 | 0.299 | -0.338 |
| Coffee | 0.021 | 0.028 | -0.328 | -0.265 | -0.017 |
| Nuts and seeds unsalted | -0.075 | -0.399 | -0.034 | -0.163 | -0.072 |
| Potatoes | 0.380 | -0.053 | 0.059 | 0.086 | 0.212 |
| Sauces and condiments | 0.217 | -0.217 | 0.003 | 0.180 | 0.267 |
| Soup | -0.038 | -0.039 | 0.312 | -0.159 | -0.119 |
| Vegetables | 0.144 | -0.441 | -0.087 | -0.111 | 0.045 |
| Legumes and beans | 0.322 | -0.087 | -0.096 | 0.010 | 0.213 |

Raw loadings of food groups per principal component (i.e., dietary pattern) in PRIMM-UK. Abbreviations: PC, principal component.

| **Food groups** | **PC1** | **PC2** | **PC3** | **PC4** | **PC5** |
|---|---|---|---|---|---|
| Alcoholic beverages | **0.489** | -0.249 | 0.107 | -0.079 | 0.253 |
| Eggs and egg dishes | **0.352** | **-0.394** | -0.226 | -0.197 | **-0.352** |
| Whole grain products | 0.234 | -0.051 | **0.544** | **0.439** | **-0.379** |
| Refined grain products | **0.361** | **0.369** | 0.277 | **-0.576** | 0.023 |
| Sweets desserts and snacks | **0.628** | 0.269 | **-0.341** | 0.242 | -0.285 |
| Fats and oils | **0.735** | 0.202 | -0.077 | 0.085 | 0.154 |
| Fish and fish products | **0.343** | **-0.388** | -0.264 | -0.181 | **-0.424** |
| Poultry | -0.162 | 0.151 | 0.209 | **0.417** | **-0.315** |
| Red and processed meat | **0.746** | **0.363** | -0.037 | -0.138 | -0.138 |
| Fruit | -0.099 | **-0.663** | 0.071 | 0.049 | -0.005 |
| Yoghurt | 0.124 | -0.054 | **0.456** | **-0.609** | 0.040 |
| Cheese | 0.205 | -0.053 | 0.257 | **0.415** | **0.415** |
| Milk | 0.131 | 0.045 | **-0.530** | 0.062 | **-0.497** |
| Fruit juice | **0.347** | 0.151 | **0.628** | **-0.403** | -0.287 |
| Sugar-sweetened beverages | 0.125 | **0.571** | -0.298 | -0.066 | 0.089 |
| Tea | **0.373** | -0.169 | **0.316** | **0.438** | **-0.450** |
| Coffee | 0.041 | 0.046 | **-0.503** | **-0.388** | -0.023 |
| Nuts and seeds unsalted | -0.145 | **-0.660** | -0.052 | -0.238 | -0.095 |
| Potatoes | **0.736** | -0.087 | 0.090 | 0.126 | 0.282 |
| Sauces and condiments | **0.420** | **-0.359** | 0.005 | 0.264 | **0.355** |
| Soup | -0.073 | -0.065 | **0.478** | -0.233 | -0.159 |
| Vegetables | 0.280 | **-0.728** | -0.133 | -0.162 | 0.060 |
| Legumes and beans | **0.625** | -0.144 | -0.148 | 0.015 | 0.284 |

**Pearson correlation coefficient for each food group with each principal component (i.e., dietary patterns) in PRIMM-UK.** Table shows the contribution of food groups to each dietary pattern. Values indicate the positive and negative loadings of food groups based on their correlation with each principal component. Values in bold indicate loadings above 0.3 (for high intakes) and below −0.3 (for low intakes, see **eFigure 4**). Abbreviations: PC, principal component.

© 2023 Bolte LA et al. *JAMA Oncology*.

eTable 6. Loadings of food groups per dietary pattern identified by PCA and their correlations in PRIMM-NL

| Food groups | **PC1** | **PC2** | **PC3** | **PC4** | **PC5** |
|---|---|---|---|---|---|
| Alcoholic beverages | 0.062 | -0.199 | 0.371 | -0.413 | 0.318 |
| Vegetables | 0.313 | -0.268 | -0.385 | 0.030 | 0.007 |
| Potatoes | 0.191 | -0.349 | -0.020 | -0.099 | 0.260 |
| Fruit | -0.101 | 0.191 | 0.084 | 0.189 | -0.267 |
| Fish | 0.003 | -0.189 | 0.384 | 0.071 | 0.094 |
| Meat | -0.091 | -0.297 | 0.037 | -0.159 | 0.296 |
| Cold cuts and spreads (fish, meat) | -0.362 | -0.194 | -0.063 | 0.110 | -0.230 |
| Sauces | 0.052 | 0.037 | -0.008 | 0.088 | 0.040 |
| Soup | 0.201 | -0.247 | -0.195 | 0.308 | 0.200 |
| Cheese | -0.023 | -0.449 | 0.187 | 0.317 | -0.056 |
| Milk and milk-based drinks | -0.195 | 0.062 | 0.247 | -0.467 | -0.099 |
| Savoury snacks and ready meals | -0.257 | -0.477 | -0.029 | 0.012 | -0.348 |
| Biscuits and cakes | -0.280 | -0.249 | -0.254 | -0.369 | -0.282 |
| Wholemeal bread | 0.392 | -0.047 | -0.200 | -0.232 | -0.317 |
| White bread | -0.418 | 0.031 | 0.051 | 0.292 | 0.279 |
| Fruit juice | -0.368 | 0.037 | -0.382 | -0.028 | 0.261 |
| Fats and oils | -0.151 | 0.084 | -0.408 | -0.207 | 0.330 |

Raw loadings of food groups per principal component (i.e., dietary pattern) in PRIMM-NL. Abbreviations: PC, principal component.

| **Food groups** | **PC1** | **PC2** | **PC3** | **PC4** | **PC5** |
|---|---|---|---|---|---|
| Alcoholic beverages | 0.102 | **-0.303** | **0.482** | **-0.519** | **0.368** |
| Vegetables | **0.514** | **-0.408** | **-0.500** | 0.038 | 0.008 |
| Potatoes | **0.314** | **-0.531** | -0.025 | -0.125 | **0.302** |
| Fruit | -0.166 | **0.291** | 0.109 | 0.237 | **-0.309** |
| Fish | 0.005 | -0.287 | **0.499** | 0.090 | 0.109 |
| Meat | -0.150 | **-0.453** | 0.048 | -0.200 | **0.343** |
| Cold cuts and spreads (fish, meat) | -0.596 | -0.296 | -0.081 | 0.138 | -0.266 |
| Sauces | 0.085 | 0.057 | -0.010 | 0.110 | 0.046 |
| Soup | **0.330** | **-0.377** | -0.253 | **0.388** | 0.231 |
| Cheese | -0.038 | **-0.685** | 0.243 | **0.399** | -0.065 |
| Milk and milk-based drinks | **-0.321** | 0.095 | **0.320** | **-0.588** | -0.115 |
| Savoury snacks and ready meals | **-0.422** | **-0.727** | -0.037 | 0.015 | **-0.403** |
| Biscuits and cakes | **-0.461** | **-0.379** | **-0.330** | **-0.464** | **-0.327** |
| Wholemeal bread | **0.645** | -0.072 | -0.259 | **-0.291** | **-0.367** |
| White bread | **-0.687** | 0.047 | 0.066 | **0.367** | **0.323** |
| Fruit juice | **-0.605** | 0.056 | **-0.497** | -0.035 | **0.302** |
| Fats and oils | -0.248 | 0.129 | **-0.530** | -0.260 | **0.382** |

**Pearson correlation coefficient for each food group with each principal component (i.e., dietary patterns) in PRIMM-NL**. Table shows the contribution of food groups to each dietary pattern. Values indicate the positive and negative loadings of food groups based on their correlation with each principal component. Values in bold indicate loadings above 0.3 (for high intakes) and below −0.3 (for low intakes, see **eFigure 4**). Abbreviations: PC, principal component.

© 2023 Bolte LA et al. *JAMA Oncology*.

eTable 7. Results of the generalized additive models using the top 5 diet PCs

| Per country: PRIMM-UK | | | | | | | **Per country: PRIMM-NL** | | | | | |
|---|---|---|---|---|---|---|---|---|---|---|---|---|
| PFS12 | | | | | | | **PFS12** | | | | | |
| variable | **edf** | **ref.df** | **Chi.sq** | **p.value** | **FDR** | | **variable** | **edf** | **ref.df** | **Chi.sq** | **p.value** | **FDR** |
| s(PC1) | 0.751 | 4 | 1.180 | 0.201 | 0.603 | | s(PC1) | 1.37E-05 | 4 | 1.01E-05 | 0.425 | 0.478 |
| s(PC2) | 0.492 | 4 | 0.901 | 0.173 | 0.192 | | s(PC2) | 2.150 | 4 | 13.400 | 0.007 | 0.018 |
| s(PC3) | 1.88E-06 | 4 | 1.01E-06 | 0.544 | 0.706 | | s(PC3) | 8.97E-06 | 4 | 7.35E-06 | 0.391 | 0.400 |
| s(PC4) | 6.87E-06 | 4 | 1.83E-06 | 0.757 | 0.757 | | s(PC4) | 0.183 | 4 | 0.234 | 0.255 | 0.383 |
| s(PC5) | 1.76E-05 | 4 | 6.79E-06 | 0.648 | 0.773 | | s(PC5) | 0.071 | 4 | 0.074 | 0.299 | 0.462 |
| s(bmi) | 2.67E-05 | 4 | 2.25E-05 | 0.397 | 0.707 | | s(bmi) | 0.087 | 4 | 0.099 | 0.278 | 0.834 |
| s(age) | 3.20E-06 | 4 | 1.29E-06 | 0.586 | 0.586 | | s(age) | 1.960 | 4 | 17.800 | 0.009 | 0.027 |
| s(sex) | 5.20E-06 | 1 | 1.05E-06 | 0.645 | 0.769 | | s(sex) | 0.884 | 1 | 5.510 | 0.010 | 0.018 |
| ORR | | | | | | | **ORR** | | | | | |
| variable | **edf** | **ref.df** | **Chi.sq** | **p.value** | **FDR** | | **variable** | **edf** | **ref.df** | **Chi.sq** | **p.value** | **FDR** |
| s(PC1) | 3.28E-06 | 4 | 1.08E-06 | 0.683 | 0.717 | | s(PC1) | 0.763 | 4 | 2.300 | 0.074 | 0.222 |
| s(PC2) | 0.444 | 4 | 0.755 | 0.192 | 0.192 | | s(PC2) | 2.700 | 4 | 20.900 | 0.012 | 0.018 |
| s(PC3) | 3.35E-06 | 4 | 2.25E-06 | 0.493 | 0.706 | | s(PC3) | 8.34E-06 | 4 | 6.44E-06 | 0.400 | 0.400 |
| s(PC4) | 5.13E-06 | 4 | 3.99E-06 | 0.447 | 0.671 | | s(PC4) | 1.77E-06 | 4 | 4.94E-08 | 0.978 | 0.978 |
| s(PC5) | 1.17E-05 | 4 | 8.19E-06 | 0.464 | 0.773 | | s(PC5) | 6.98E-06 | 4 | 4.30E-06 | 0.462 | 0.462 |
| s(bmi) | 6.36E-06 | 4 | 4.38E-06 | 0.471 | 0.707 | | s(bmi) | 3.61E-06 | 4 | 8.45E-07 | 0.770 | 0.961 |
| s(age) | 6.43E-07 | 4 | 3.05E-07 | 0.571 | 0.586 | | s(age) | 1.800 | 4 | 7.900 | 0.044 | 0.066 |
| s(sex) | 2.21E-06 | 1 | 5.95E-07 | 0.595 | 0.769 | | s(sex) | 0.880 | 1 | 5.300 | 0.012 | 0.018 |
| irAEs | | | | | | | **irAEs** | | | | | |
| variable | **edf** | **ref.df** | **Chi.sq** | **p.value** | **FDR** | | **variable** | **edf** | **ref.df** | **Chi.sq** | **p.value** | **FDR** |
| s(PC1) | 9.50E-06 | 4 | 2.72E-06 | 0.717 | 0.717 | | s(PC1) | 7.09E-06 | 4 | 4.45E-06 | 0.478 | 0.478 |
| s(PC2) | 1.750 | 4 | 4.760 | 0.055 | 0.165 | | s(PC2) | 0.987 | 4 | 1.600 | 0.184 | 0.184 |
| s(PC3) | 5.89E-06 | 4 | 1.89E-06 | 0.706 | 0.706 | | s(PC3) | 0.914 | 4 | 1.610 | 0.160 | 0.400 |
| s(PC4) | 2.39E-05 | 4 | 1.84E-05 | 0.426 | 0.671 | | s(PC4) | 0.837 | 4 | 4.080 | 0.025 | 0.075 |
| s(PC5) | 6.76E-06 | 4 | 1.67E-06 | 0.773 | 0.773 | | s(PC5) | 1.19E-05 | 4 | 9.97E-06 | 0.374 | 0.462 |
| s(bmi) | 4.57E-06 | 4 | 1.52E-07 | 0.977 | 0.977 | | s(bmi) | 3.21E-06 | 4 | 1.52E-07 | 0.961 | 0.961 |
| s(age) | 1.290 | 4 | 6.420 | 0.009 | 0.027 | | s(age) | 0.303 | 4 | 0.353 | 0.294 | 0.294 |
| s(sex) | 3.67E-06 | 1 | 3.10E-07 | 0.769 | 0.769 | | s(sex) | 2.98E-06 | 1 | 1.34E-06 | 0.484 | 0.484 |

**eTable7. Results of the generalized additive models using the top 5 diet PCs.** Principal component analysis (PCA) was performed per cohort to identify country-specific dietary patterns. Logistic generalized additive models (GAMs) were used to determine whether a higher adherence to the identified dietary patterns is associated with a higher probability of response or irAEs. In each cohort, we modelled each outcome variable (ORR, PFS-12, irAEs) and the first 5 principal components as independent variables adjusting for age, sex, and BMI. Abbreviations: PFS12, Progression-free survival at 12 months; ORR, Overall response rate; irAEs, immune-related adverse events; PC, principal component

© 2023 I

eTable 8. Results of the generalized additive models using nutrients

| | | | | | | | | | | | | |
|---|---|---|---|---|---|---|---|---|---|---|---|---|
| PFS12 | | | | | | | **PFS12** | | | | | |
| term | **edf** | **ref.df** | **Chi.sq** | **p.value** | **FDR** | | **term** | **edf** | **ref.df** | **Chi.sq** | **p.value** | **FDR** |
| s(Beta_carotene_mcg) | 7.96E-01 | 4 | 3.435 | 0.037 | 0.276 | | **s(Vitamin_C_ ascorbic_acid_mg)** | 1.725 | 4 | 6.110 | 0.022 | 0.238 |
| s(bmi) | 5.10E-01 | 4 | 0.690 | 0.247 | | | s(bmi) | 1.59E-05 | 4 | 1.16E-05 | 0.436 | |
| s(age) | 9.86E-07 | 4 | 6.64E-08 | 1.000 | | | s(age) | 2.25E-06 | 4 | 1.11E-06 | 0.546 | |
| s(sex) | 4.96E-01 | 1 | 0.915 | 0.173 | | | s(sex) | 2.69E-06 | 1 | 8.05E-07 | 0.581 | |
| s(Energy_kcal) | 1.05E-04 | 4 | 8.95E-05 | 0.381 | | | s(Energy_kcal) | 0.784 | 4 | 1.274 | 0.186 | |
| | | | | | | | | | | | | |
| ORR | | | | | | | **ORR** | | | | | |
| term | **edf** | **ref.df** | **Chi.sq** | **p.value** | **FDR** | | **term** | **edf** | **ref.df** | **Chi.sq** | **p.value** | **FDR** |
| s(Beta_carotene_mcg) | 8.13E-01 | 4 | 3.820 | 0.030 | 0.320 | | **s(Vitamin_C_ ascorbic_acid_mg)** | 1.595 | 4 | 5.353 | 0.032 | 0.320 |
| s(bmi) | 3.06E-05 | 4 | 2.58E-05 | 0.395 | | | s(bmi) | 9.44E-06 | 4 | 5.17E-06 | 0.526 | |
| s(age) | 1.10E-06 | 4 | 8.75E-08 | 1.000 | | | s(age) | 8.39E-07 | 4 | 3.41E-07 | 0.601 | |
| s(sex) | 4.29E-01 | 1 | 0.706 | 0.199 | | | s(sex) | 2.39E-06 | 1 | 5.62E-07 | 0.627 | |
| s(Energy_kcal) | 2.04E-05 | 4 | 1.69E-05 | 0.381 | | | s(Energy_kcal) | 7.84E-06 | 4 | 2.58E-06 | 0.698 | |
| | | | | | | | | | | | | |
| PFS12 | | | | | | | **PFS12** | | | | | |
| term | **edf** | **ref.df** | **Chi.sq** | **p.value** | **FDR** | | **term** | **edf** | **ref.df** | **Chi.sq** | **p.value** | **FDR** |
| s(PUFA_total_g) | 0.879 | 4 | 6.507 | 0.008 | 0.238 | | **s(MUFA_total_g)** | 0.738 | 4 | 2.617 | 0.024 | 0.238 |
| s(bmi) | 3.83E-05 | 4 | 3.12E-05 | 0.407 | | | s(bmi) | 1.29E-05 | 4 | 6.12E-06 | 0.565 | |
| s(age) | 2.30E-06 | 4 | 1.31E-06 | 0.507 | | | s(age) | 3.38E-06 | 4 | 1.04E-06 | 0.678 | |
| s(sex) | 0.426 | 1 | 0.687 | 0.203 | | | s(sex) | 0.583 | 1 | 1.286 | 0.135 | |
| s(Energy_kcal) | 9.84E-06 | 4 | 6.06E-06 | 0.513 | | | s(Energy_kcal) | 0.526 | 4 | 0.691 | 0.199 | |

© 2023 Bolte LA et al. *JAMA Oncology*.

| | | | | | | | | | | | | |
|---|---|---|---|---|---|---|---|---|---|---|---|---|
| ORR | | | | | | | **PFS12** | | | | | |
| term | **edf** | **ref.df** | **Chi.sq** | **p.value** | **FDR** | | **term** | **edf** | **ref.df** | **Chi.sq** | **p.value** | **FDR** |
| s(PUFA_total_g) | 0.822 | 4 | 4.052 | 0.028 | 0.320 | | **s(Vitamin_E_α_ tocopherol _equivalents_mg)** | 1.311 | 4 | 3.798 | 0.054 | 0.298 |
| s(bmi) | 9.92E-06 | 4 | 2.71E-06 | 0.745 | | | s(bmi) | 7.67E-06 | 4 | 2.48E-06 | 0.684 | |
| s(age) | 1.01E-05 | 4 | 8.19E-06 | 0.398 | | | s(age) | 7.95E-06 | 4 | 2.99E-06 | 0.625 | |
| s(sex) | 0.313 | 1 | 0.426 | 0.242 | | | s(sex) | 0.167 | 1 | 0.197 | 0.276 | |
| s(Energy_kcal) | 1.46E-05 | 4 | 9.47E-06 | 0.481 | | | s(Energy_kcal) | 1.13E-05 | 4 | 4.34E-06 | 0.620 | |
| | | | | | | | | | | | | |
| irAEs | | | | | | | **irAEs** | | | | | |
| term | **edf** | **ref.df** | **Chi.sq** | **p.value** | **FDR** | | **term** | **edf** | **ref.df** | **Chi.sq** | **p.value** | **FDR** |
| s(Beta_carotene_mcg) | 3.201 | 4 | 8.297 | 0.038 | 0.361 | | **s(Magnesium_mg)** | 1.499 | 4 | 5.066 | 0.016 | 0.361 |
| s(bmi) | 0.305 | 4 | 0.416 | 0.242 | | | s(bmi) | 1.28E-05 | 4 | 1.23E-06 | 1.000 | |
| s(age) | 0.842 | 4 | 4.250 | 0.024 | | | s(age) | 1.827 | 4 | 5.371 | 0.043 | |
| s(sex) | 2.78E-06 | 1 | 1.02E-07 | 0.846 | | | s(sex) | 8.53E-06 | 1 | 5.61E-09 | 0.979 | |
| s(Energy_kcal) | 7.11E-06 | 4 | 2.33E-06 | 0.632 | | | s(Energy_kcal) | 1.253 | 4 | 2.836 | 0.075 | |
| | | | | | | | | | | | | |

**eTable8.** Results of the generalized additive models using nutrients. Nutrients were analysed using the FFQ EPIC tool for analysis (FETA) in PRIMM-UK (doi:10.1136/bmjopen-2013-004503). Logistic generalized additive models (GAMs) were used to assess the relationship between specific food groups and nutrients and response or irAEs, constructing one model for each food group or nutrient. Abbreviations: PFS12, Progression-free survival at 12 months; ORR, Overall response rate; irAEs, immune-related adverse events; bmi, body mass index; mcg, micrograms; mg, milligrams; g, grams.

© 2023 Bolte LA et al. *JAMA Oncology*.

eTable 9. Results of the generalized additive models using food groups per country

Per country: PRIMM-UK

| | | | | | |
|---|---|---|---|---|---|
| PFS12 | | | | | |
| term | **edf** | **ref.df** | **Chi.sq** | **p.value** | **FDR** |
| s(Vegetables) | 1.390 | 4 | 4.430 | 0.040 | 0.347 |
| s(Energy_kcal) | 7.81E-06 | 4 | 2.71E-06 | 0.658 | |
| s(bmi) | 9.57E-06 | 4 | 4.70E-06 | 0.581 | |
| s(age) | 8.47E-06 | 4 | 2.72E-06 | 0.683 | |
| s(sex) | 6.44E-06 | 1 | 4.69E-06 | 0.391 | |
| | | | | | |
| ORR | | | | | |
| term | **edf** | **ref.df** | **Chi.sq** | **p.value** | **FDR** |
| s(Nuts.and.seeds.unsalted) | 1.540 | 4 | 3.760 | 0.083 | 0.604 |
| s(Energy_kcal) | 9.56E-06 | 4 | 5.93E-06 | 0.513 | |
| s(bmi) | 1.06E-05 | 4 | 4.05E-06 | 0.647 | |
| s(age) | 2.91E-06 | 4 | 4.38E-07 | 0.819 | |
| s(sex) | 5.99E-06 | 1 | 3.49E-06 | 0.445 | |
| | | | | | |
| PFS12 | | | | | |
| term | **edf** | **ref.df** | **Chi.sq** | **p.value** | **FDR** |
| s(Yoghurt) | 0.713 | 4 | 2.260 | 0.075 | 0.347 |
| s(Energy_kcal) | 4.72E-05 | 4 | 4.27E-05 | 0.398 | |
| s(bmi) | 1.30E-05 | 4 | 5.56E-06 | 0.615 | |
| s(age) | 5.55E-06 | 4 | 2.50E-06 | 0.513 | |
| s(sex) | 0.240 | 1 | 0.313 | 0.253 | |
| | | | | | |
| PFS12 | | | | | |
| term | **edf** | **ref.df** | **Chi.sq** | **p.value** | **FDR** |
| s(Legumes.and.beans) | 0.755 | 4 | 2.746 | 0.057 | 0.347 |
| s(Energy_kcal) | 2.07E-05 | 4 | 0.423 | 0.290 | |
| s(bmi) | 1.32E-05 | 4 | 2.32E-06 | 0.723 | |
| s(age) | 8.89E-07 | 4 | 4.48E-07 | 0.565 | |
| s(sex) | 0.152 | 1 | 1.070 | 0.150 | |

**Per country: PRIMM-NL**

| | | | | | |
|---|---|---|---|---|---|
| **ORR** | | | | | |
| **term** | **edf** | **ref.df** | **Chi.sq** | **p.value** | **FDR** |
| **s(Vegetables)** | 0.796 | 4 | 3.730 | 0.0402 | 0.148 |
| s(bmi) | 1.80E-05 | 4 | 0.000 | 0.573 | |
| s(age) | 1.540 | 4 | 3.660 | 0.127 | |
| s(sex) | 0.800 | 1 | 3.430 | 0.0365 | |
| | | | | | |
| | | | | | |
| **ORR** | | | | | |
| **term** | **edf** | **ref.df** | **Chi.sq** | **p.value** | **FDR** |
| **s(Potatoes)** | 0.868 | 4 | 10.072 | 0.0135 | 0.108 |
| s(bmi) | 1.44E-05 | 4 | 0.000 | 0.70847 | |
| s(age) | 0.400 | 4 | 2.962 | 0.16044 | |
| s(sex) | 0.895 | 1 | 7.138 | 0.00398 | |
| | | | | | |
| | | | | | |
| **ORR** | | | | | |
| **term** | **edf** | **ref.df** | **Chi.sq** | **p.value** | **FDR** |
| **s(Wholemeal.bread)** | 1.195 | 4 | 4.437 | 0.0463 | 0.148 |
| s(bmi) | 1.01E-05 | 4 | 0.000 | 0.9645 | |
| s(age) | 1.155 | 4 | 1.878 | 0.2311 | |
| s(sex) | 0.846 | 1 | 4.677 | 0.017 | |
| | | | | | |
| | | | | | |
| **PFS12** | | | | | |
| **term** | **edf** | **ref.df** | **Chi.sq** | **p.value** | **FDR** |
| **s(Wholemeal.bread)** | 1.681 | 4 | 5.373 | 0.0488 | 0.196 |
| s(bmi) | 0.442 | 4 | 0.748 | 0.1912 | |
| s(age) | 0.947 | 4 | 3.586 | 0.1021 | |
| s(sex) | 0.855 | 1 | 4.839 | 0.015 | |

© 2023 Bolte LA et al. *JAMA Oncology*.

| | | | | | | | | | | | | |
|---|---|---|---|---|---|---|---|---|---|---|---|---|
| Per country: PRIMM-UK | | | | | | | **Per country: PRIMM-NL** | | | | | |
| | | | | | | | | | | | | |
| irAEs | | | | | | | **irAEs** | | | | | |
| term | **edf** | **ref.df** | **Chi.sq** | **p.value** | **FDR** | | **term** | **edf** | **ref.df** | **Chi.sq** | **p.value** | **FDR** |
| s(Processed.Meat) | 0.847 | 4 | 4.383 | 0.020 | 0.261 | | **s(Fruit)** | 0.798 | 2 | 3.834 | 0.0367 | 0.587 |
| s(Energy_kcal) | 0.805 | 4 | 0.000 | 0.071 | | | s(bmi) | 7.04E-06 | 4 | 0.000 | 0.7598 | |
| s(bmi) | 1.02E-05 | 4 | 0.000 | 0.988 | | | s(age) | 0.456 | 4 | 0.671 | 0.2442 | |
| s(age) | 1.638 | 4 | 0.000 | 0.033 | | | s(sex) | 0.761 | 1 | 2.897 | 0.0479 | |
| s(sex) | 4.93E-06 | 1 | 0.177 | 0.852 | | | | | | | | |
| | | | | | | | | | | | | |
| irAEs | | | | | | | | | | | | |
| term | **edf** | **ref.df** | **Chi.sq** | **p.value** | **FDR** | | | | | | | |
| s(Whole.grain.products) | 0.841 | 4 | 4.690 | 0.018 | 0.203 | | | | | | | |
| s(Energy_kcal) | 1.22E-05 | 4 | 0.000 | 0.743 | | | | | | | | |
| s(bmi) | 6.77E-05 | 4 | 0.000 | 0.349 | | | | | | | | |
| s(age) | 5.19E-05 | 4 | 0.000 | 0.363 | | | | | | | | |
| s(sex) | 5.84E-06 | 1 | 0.000 | 0.627 | | | | | | | | |
| | | | | | | | | | | | | |
| irAEs | | | | | | | | | | | | |
| term | **edf** | **ref.df** | **Chi.sq** | **p.value** | **FDR** | | | | | | | |
| s(Legumes.and.beans) | 0.752 | 4 | 2.725 | 0.052 | 0.261 | | | | | | | |
| s(Energy_kcal) | 0.517 | 4 | 0.643 | 0.279 | | | | | | | | |
| s(bmi) | 6.24E-06 | 4 | 0.000 | 0.777 | | | | | | | | |
| s(age) | 0.773 | 4 | 2.874 | 0.052 | | | | | | | | |
| s(sex) | 4.01E-06 | 1 | 0.000 | 0.741 | | | | | | | | |
| eTable9. Results of the generalized additive models using individual food groups per country. Logistic generalized additive models (GAMs) were used to assess the relationship between specific food groups and response or irAEs, constructing one model for each food group. Abbreviations: PFS12, Progression-free survival at 12 months; ORR, Overall response rate; irAEs, immune-related adverse events; kcal, kilocalories; bmi, body mass index; kcal, kilocalories. | | | | | | | | | | | | |

© 2023 Bolte LA et al. *JAMA Oncology*.

**eTable 10. Overview of literature linking cancer, nutrition, and immune checkpoint blockade**

| Study | Study Type | Methods | Findings | Dietary factors |
|---|---|---|---|---|
| [12] Sivan et al. | Pre-clinical | Firstly, used 16S sequencing of mouse stool following ICB treatment to identify *microbial strains* associated with anti-tumor effects. Then used *Bifidobacterium* as a probiotic in melanoma-model mice. | Orally administered *Bifidobacterium* improved tumor control in mice to the same degree as PD-L1 ICB; Combination of *Bifidobacterium* and PD-L1 ICB nearly abolished tumor growth. | High fiber<br>High in prebiotics<br>Whole grains<br>Fermented foods |
| [13] Yuan et al. | Observational; Pre-clinical | Retrospective analysis of electronic health records to link supplement use to clinical outcomes in patients treated with ICB and subsequent murine model to investigate whether Vitamin E increased anti-tumor efficacy in mice bearing EMT6 mammary tumors | ICB-treated cancer patients who took vitamin E had significantly improved survival which was confirmed in a murine model and mediated by invigorating dendritic cells | Vitamin E |
| [14] Magrì et al. | Pre-clinical | Investigated whether the combination with high dose vitamin C could enhance efficacy of ICB in mice bearing syngeneic pancreatic, breast, or colorectal tumors | Addition of vitamin C enhanced efficacy of ICB in several tumor types with an increased cytotoxic activity of adoptively transferred CD8 T cells and recruitment of T lymphocytes in the tumor microenvironment. | High dose vitamin C |
| [15] Huang et al. | Pre-clinical | Investigated whether the combination with ginseng polysaccharides (GPs) could potentiate the response to anti-PD-1/PD-L1 antibodies by modulating gut microbiota in syngeneic mouse models. | In mice inoculated with lung cancer, the combination of GPs with anti-PD1 antibody treatment increased the anti-tumor response by causing an increase in valerate and a decrease in kynurenine and the kynurenine: tryptophan ratio. | Ginseng polysaccharides |
| [16] Wang et al. | Pre-clinical | Combined anti–CTLA-4 antibody treatment with a standard colitis model to give mice severe ICB-induced colitis before administering Bifidobacterium as a probiotic. | *Bifidobacterium* largely rescued mice from immunopathology | High fiber<br>High in prebiotics<br>Whole grains<br>Fermented foods |
| [17] Vétizou et al. | Pre-clinical | Investigated T cell responses specific for *B. thetaiotaomicron* or *B. fragilis* and CTLA-4 blockade | Tumors in antibiotic-treated or germ-free mice did not respond to anti-CTLA-4. This defect was overcome by gavage with *B. fragilis*, by immunization with *B. fragilis* polysaccharides, or by adoptive transfer of *B. fragilis*–specific T cells. T cell responses specific for *B. thetaiotaomicron* | Soluble fibers |

© 2023 Bolte LA et al. *JAMA Oncology*.

| Study | Study Type | Methods | Findings | Dietary factors |
|---|---|---|---|---|
| | | | or *B. fragilis* were associated with anti-CTLA-4 efficacy in mice | |
| **Study** | **Study Type** | **Methods** | **Findings** | **Dietary factors** |
| [18] Ferrere et al. | Pre-clinical | Investigated the functional impact of KD and ketone bodies alone (3HB) on the immune system, tumor surveillance, and gut microbiota in mice inoculated with melanoma (RET) or renal cancer (RENCA) and treated with anti-PDL-1 and anti-CTLA-4 antibodies. | In conditions in which anti-PD-1 antibody alone or plus anti-CTLA-4 antibody failed to reduce tumor growth in mice receiving a normal diet, KD, or 3HB supplementation reestablished therapeutic responses. KD led to increases in *Eisenbergiella massilience* and *Akkermansia muciniphila* | Ketogenic diet;<br>Ketone bodies |
| [19] Spencer et al. | Observational | Pre-treatment fiber intake was estimated from dietary screeners and linked to response, PFS and taxonomic abundance in melanoma patients treated with ICB. | High fiber intake was associated with response to ICB, while the opposite was found for commercially available probiotics. The role of a high- fiber diet was confirmed in conventionally housed SPF- but not in germ-free mice. | Commercially available probiotics and fiber intake estimated from dietary screeners completed prior to treatment |
| [20] Nomura et al. | Observational; pre-clinical | Patients receiving ICB were classified into 2 groups based on their treatment response. Dietary information was obtained. SCFA-concentrations in stool and plasma collected before ICB initiation were measured using ultra-high-performance liquid chromatography coupled with tandem mass spectrometry. | In this cohort study of 52 patients with solid tumors, high concentrations of fecal acetic acid, propionic acid, butyric acid, and valeric acid were significantly associated with longer progression-free survival. Of the dietary items assessed, mushroom intake was associated with longer progression-free survival. | Mushroom intake assessed by an FFQ completed 1 year prior to treatment |
| [21] Simpson et al. | Observational | Pre-treatment fiber and omega-3 fatty acid intake was estimated from FFQs and linked to response and taxonomic abundance in Stage 3 melanoma patients treated with ICB. | Low intake of omega 3 fatty acids was associated with non-response and adverse outcomes. Omega 3 consumption was positively associated with microbial diversity and butyrate producing microbial pathways. Integration of data from Spencer et al [19] confirmed the role of fiber for improved response parameters. | Omega-3 fatty acids,<br>fiber |

Overview of preclinical and observational studies investigating the potential of diet to enhance response to immune checkpoint blockade. Column *Dietary factors* shows diets, foods and nutrients associated with abundance of relevant bacteria and ICB-response. Abbreviations: PD-L1, programmed death receptor ligand-1; CTLA-4, cytotoxic T-lymphocyte associated protein-4; ICB, immune checkpoint blockade; RET, Ret proto-oncogene; FFQ, food frequency questionnaires; SCFAs, short chain fatty acids.

© 2023 Bolte LA et al. *JAMA Oncology*.

eTable 11. Description of Outliers

| Patient ID | **MVCC001** | **MVCC006** | **MVCC011** | **MVCC012** | **MVCC018** | **MVCC024** | **MVCC041** | **MVCC052** | **MVCC054** |
|---|---|---|---|---|---|---|---|---|---|
| Centre | PRIMM-UK | PRIMM-UK | PRIMM-UK | PRIMM-UK | PRIMM-UK | PRIMM-UK | PRIMM-UK | PRIMM-UK | PRIMM-UK |
| ANTHROPOMETRIC DATA | | | | | | | | | |
| Age | 53 | 83 | 86 | 19 | 46 | 56 | 46 | 84 | 55 |
| Sex | female | male | male | male | female | male | male | male | male |
| Weight | 72.3 | 83.4 | 114 | 67.5 | 64.2 | 98 | 74.2 | 81.2 | 80.2 |
| Height | 167 | 173 | 183 | 176 | 164 | 177 | 175 | 167 | 179.5 |
| BMI | 25.92 | 27.87 | 34.04 | 21.79 | 23.87 | 31.28 | 24.23 | 29.12 | 24.89 |
| Energy (kcal) | 8903.68 | 3299.19 | 2805.54 | 4313.79 | 4091.02 | 693.74 | 680.24 | 3384.87 | 1895.36 |
| median (kcal) | 1726.85 | 1726.85 | 1726.85 | 1726.85 | 1726.85 | 1726.85 | 1726.85 | 1726.85 | 1726.85 |
| mean ±SD (kcal) | 2057.94 ± 1333.51 | 2057.94 ± 1333.51 | 2057.94 ± 1333.51 | 2057.94 ± 1333.51 | 2057.94 ± 1333.51 | 2057.94 ± 1333.51 | 2057.94 ± 1333.51 | 2057.94 ± 1333.51 | 2057.94 ± 1333.51 |
| BMR | 1414.76 | 1512.47 | 1968.47 | 1753.22 | 1365.44 | 1920.97 | 1648.47 | 1448.22 | 1695.22 |
| EI/BMR ratio | 6.29 | 2.18 | 1.43 | 2.46 | 3 | 0.36 | 0.41 | 2.34 | 1.12 |
| Missing ticks FFQ | 0 | 0 | 2 | 17 | 1 | 0 | 30 | 0 | 2 |
| MACRONUTRIENT INTAKE | | | | | | | | | |
| Alcohol (g) | 0 | 3.11 | 5.98 | 21.77 | 28.49 | 4.87 | 4.87 | 9.4 | 5.47 |
| median (g) | 5.47 | 5.47 | 5.47 | 5.47 | 5.47 | 5.47 | 5.47 | 5.47 | 5.47 |
| mean ±SD (g) | 7.52 ±8.31 | 7.52 ±8.31 | 7.52 ±8.31 | 7.52 ±8.31 | 7.52 ±8.31 | 7.52 ±8.31 | 7.52 ±8.31 | 7.52 ±8.31 | 7.52 ±8.31 |
| Carbohydrate (total g) | 1294.89 | 394.51 | 292.01 | 264.83 | 434.41 | 114.84 | 76 | 408.13 | 155.5 |
| median (g) | 189 | 189 | 189 | 189 | 189 | 189 | 189 | 189 | 189 |
| mean ±SD (g) | 230.83 ± 181.23 | 230.83 ± 181.23 | 230.83 ± 181.23 | 230.83 ± 181.23 | 230.83 ± 181.23 | 230.83 ± 181.23 | 230.83 ± 181.23 | 230.83 ± 181.23 | 230.83 ± 181.23 |
| Protein (g) | 198.88 | 137.92 | 126.34 | 244.93 | 173.82 | 22.4 | 29.22 | 153.79 | 175.97 |
| median (g) | 75.93 | 75.93 | 75.93 | 75.93 | 75.93 | 75.93 | 75.93 | 75.93 | 75.93 |
| mean ±SD (g) | 89.52 ±44.94 | 89.52 ±44.94 | 89.52 ±44.94 | 89.52 ±44.94 | 89.52 ±44.94 | 89.52 ±44.94 | 89.52 ±44.94 | 89.52 ±44.94 | 89.52 ±44.94 |
| Fat (total g) | 361.24 | 139.16 | 129.21 | 243.53 | 174.19 | 15.91 | 27.44 | 131.15 | 63.96 |
| median (g) | 66.43 | 66.43 | 66.43 | 66.43 | 66.43 | 66.43 | 66.43 | 66.43 | 66.43 |
| mean ±SD (g) | 87.11 ±60.25 | 87.11 ±60.25 | 87.11 ±60.25 | 87.11 ±60.25 | 87.11 ±60.25 | 87.11 ±60.25 | 87.11 ±60.25 | 87.11 ±60.25 | 87.11 ±60.25 |
| | | | | | | | | | |

© 2023 Bolte LA et al. *JAMA Oncology*.

| | | | | | | | | | |
|---|---|---|---|---|---|---|---|---|---|
| Patient ID | **MVCC001** | **MVCC006** | **MVCC011** | **MVCC012** | **MVCC018** | **MVCC024** | **MVCC041** | **MVCC052** | **MVCC054** |
| Centre | PRIMM-UK | PRIMM-UK | PRIMM-UK | PRIMM-UK | PRIMM-UK | PRIMM-UK | PRIMM-UK | PRIMM-UK | PRIMM-UK |
| PRINCIPAL COMPONENT SCORES (DIETATY PATTERNS) | | | | | | | | | |
| PC1 | 10.49 | 2.27 | 0.71 | 5.25 | 3.75 | -0.84 | -1.74 | 2.92 | -2.76 |
| median | -0.802 | -0.802 | -0.802 | -0.802 | -0.802 | -0.802 | -0.802 | -0.802 | -0.802 |
| mean ±SD | 1.34e-15 ±2.27 | 1.34e-15 ±2.27 | 1.34e-15 ±2.27 | 1.34e-15 ±2.27 | 1.34e-15 ±2.27 | 1.34e-15 ±2.27 | 1.34e-15 ±2.27 | 1.34e-15 ±2.27 | 1.34e-15 ±2.27 |
| PC2 | 6.22 | 0.21 | -3.37 | -6.98 | -3.34 | 0.14 | -0.33 | 0.81 | 1.46 |
| median | 0.06 | 0.06 | 0.06 | 0.06 | 0.06 | 0.06 | 0.06 | 0.06 | 0.06 |
| mean ±SD | 2.14e-16 ±1.73 | 2.14e-16 ±1.73 | 2.14e-16 ±1.73 | 2.14e-16 ±1.73 | 2.14e-16 ±1.73 | 2.14e-16 ±1.73 | 2.14e-16 ±1.73 | 2.14e-16 ±1.73 | 2.14e-16 ±1.73 |
| PC3 | 1.35 | -1.15 | 0.78 | -0.46 | -0.64 | -1 | -0.71 | -4.97 | -4.44 |
| median | 0.212 | 0.212 | 0.212 | 0.212 | 0.212 | 0.212 | 0.212 | 0.212 | 0.212 |
| mean ±SD | -8.85e-18 ±1.37 | -8.85e-18 ± 1.37 | -8.85e-18 ±1.37 | -8.85e-18 ±1.37 | -8.85e-18 ±1.37 | -8.85e-18 ±1.37 | -8.85e-18 ±1.37 | -8.85e-18 ±1.37 | -8.85e-18 ±1.37 |
| PC4 | -0.06 | -1.09 | -2.06 | -0.85 | 1.38 | -0.07 | 0.63 | 3.4 | -1.18 |
| median | -0.003 | -0.003 | -0.003 | -0.003 | -0.003 | -0.003 | -0.003 | -0.003 | -0.003 |
| mean ±SD | 7.54e-19 ±1.31 | 7.54e-19 ±1.31 | 7.54e-19 ±1.31 | 7.54e-19 ±1.31 | 7.54e-19 ±1.31 | 7.54e-19 ±1.31 | 7.54e-19 ±1.31 | 7.54e-19 ±1.31 | 7.54e-19 ±1.31 |
| PC5 | 0.28 | 1.22 | -0.85 | 0.47 | 0.82 | 1.22 | 0.91 | -3 | 2.86 |
| median | 0.08 | 0.08 | 0.08 | 0.08 | 0.08 | 0.08 | 0.08 | 0.08 | 0.08 |
| mean ±SD | 1.28e-16 ±1.30 | 1.28e-16 ±1.30 | 1.28e-16 ±1.30 | 1.28e-16 ±1.30 | 1.28e-16 ±1.30 | 1.28e-16 ±1.30 | 1.28e-16 ±1.30 | 1.28e-16 ±1.30 | 1.28e-16 ±1.30 |
| | | | | | | | | | |
| FOOD GROUP INTAKE | | | | | | | | | |
| Alcoholic beverages (g) | 0 | 75.32 | 93.02 | 487.74 | 482.69 | 123.84 | 123.84 | 157.71 | 91.41 |
| median (g) | 93.02 | 93.02 | 93.02 | 93.02 | 93.02 | 93.02 | 93.02 | 93.02 | 93.02 |
| mean ±SD (g) | 158.7 5 ±185.84 | 158.75 ±185.84 | 158.75 ±185.84 | 158.75 ±185.84 | 158.75 ±185.84 | 158.75 ±185.84 | 158.75 ±185.84 | 158.75 ±185.84 | 158.75 ±185.84 |
| Eggs and egg dishes (g) | 50 | 21.5 | 3.5 | 0 | 3.5 | 3.5 | 0 | 32 | 3.5 |
| median (g) | 21.5 | 21.5 | 21.5 | 21.5 | 21.5 | 21.5 | 21.5 | 21.5 | 21.5 |
| mean ±SD (g) | 20.23 ±15.18 | 20.23 ±15.18 | 20.23 ±15.18 | 20.23 ±15.18 | 20.23 ±15.18 | 20.23 ±15.18 | 20.23 ±15.18 | 20.23 ±15.18 | 20.23 ±15.18 |

© 2023 Bolte LA et al. *JAMA Oncology*.

| | | | | | | | | | |
|---|---|---|---|---|---|---|---|---|---|
| Patient ID | **MVCC001** | **MVCC006** | **MVCC011** | **MVCC012** | **MVCC018** | **MVCC024** | **MVCC041** | **MVCC052** | **MVCC054** |
| Centre | PRIMM-UK | PRIMM-UK | PRIMM-UK | PRIMM-UK | PRIMM-UK | PRIMM-UK | PRIMM-UK | PRIMM-UK | PRIMM-UK |
| Whole grain products (g) | 162.5 | 177.45 | 54.55 | 51.35 | 161.3 | 65 | 0 | 162.5 | 684 |
| median (g) | 65.03 | 65.03 | 65.03 | 65.03 | 65.03 | 65.03 | 65.03 | 65.03 | 65.03 |
| mean ±SD (g) | 95.65 ±112.56 | 95.65 ±112.56 | 95.65 ±112.56 | 95.65 ± 112.56 | 95.65 ± 112.56 | 95.65 ±112.56 | 95.65 ± 112.56 | 95.65 ±112.56 | 95.65 ± 112.56 |
| Refined grain products (g) | 527.5 | 17.9 | 71.9 | 51.5 | 262 | 42 | 30 | 212.2 | 10.5 |
| median (g) | 60.8 | 60.8 | 60.8 | 60.8 | 60.8 | 60.8 | 60.8 | 60.8 | 60.8 |
| mean ±SD (g) | 86 ±87.35 | 86 ±87.35 | 86 ±87.35 | 86 ±87.35 | 86 ±87.35 | 86 ±87.35 | 86 ±87.35 | 86 ±87.35 | 86 ±87.35 |
| Sweets desserts and snacks (g) | 1112.7 | 403.52 | 415.81 | 128.53 | 138.58 | 17.46 | 8.82 | 166.93 | 50.62 |
| median (g) | 95.15 | 95.15 | 95.15 | 95.15 | 95.15 | 95.15 | 95.15 | 95.15 | 95.15 |
| mean ±SD (g) | 141.18 ±175.84 | 141.18 ±175.84 | 141.18 ±175.84 | 141.18 ±175.84 | 141.18 ±175.84 | 141.18 ±175.84 | 141.18 ±175.84 | 141.18 ±175.84 | 141.18 ±175.84 |
| Fats and oils (g) | 45 | 25 | 10 | 60.2 | 48.5 | 2.1 | 10 | 26.4 | 0.7 |
| median (g) | 10 | 10 | 10 | 10 | 10 | 10 | 10 | 10 | 10 |
| mean ±SD (g) | 17.76 ±16.15 | 17.76 ±16.15 | 17.76 ±16.15 | 17.76 ±16.15 | 17.76 ±16.15 | 17.76 ±16.15 | 17.76 ±16.15 | 17.76 ±16.15 | 17.76 ±16.15 |
| Fish and fish products (g) | 66.85 | 81.36 | 121.73 | 389.74 | 125.5 | 0 | 0 | 51.8 | 0 |
| median (g) | 40.46 | 40.46 | 40.46 | 40.46 | 40.46 | 40.46 | 40.46 | 40.46 | 40.46 |
| mean ±SD (g) | 55.8 ±63.37 | 55.8 ±63.37 | 55.8 ±63.37 | 55.8 ±63.37 | 55.8 ±63.37 | 55.8 ±63.37 | 55.8 ±63.37 | 55.8 ±63.37 | 55.8 ±63.37 |
| Poultry (g) | 16.1 | 49.45 | 49.45 | 16.1 | 115 | 8.05 | 16.1 | 49.45 | 517.5 |
| median (g) | 16.1 | 16.1 | 16.1 | 16.1 | 16.1 | 16.1 | 16.1 | 16.1 | 16.1 |
| mean ±SD (g) | 42.75 ±74.49 | 42.75 ±74.49 | 42.75 ±74.49 | 42.75 ±74.49 | 42.75 ±74.49 | 42.75 ±74.49 | 42.75 ±74.49 | 42.75 ±74.49 | 42.75 ±74.49 |
| Red and processed meat (g) | 87.83 | 180.32 | 138.76 | 506.74 | 292.63 | 10.22 | 27.98 | 247.52 | 15.26 |
| median (g) | 82.23 | 82.23 | 82.23 | 82.23 | 82.23 | 82.23 | 82.23 | 82.23 | 82.23 |
| mean ±SD (g) | 98.12 ±90.25 | 98.12 ±90.25 | 98.12 ±90.25 | 98.12 ±90.25 | 98.12 ±90.25 | 98.12 ±90.25 | 98.12 ±90.25 | 98.12 ±90.25 | 98.12 ±90.25 |
| Fruit (g) | 899.6 | 189.35 | 107.95 | 128.25 | 467.95 | 315 | 116.1 | 81.7 | 17.2 |
| median (g) | 173.95 | 173.95 | 173.95 | 173.95 | 173.95 | 173.95 | 173.95 | 173.95 | 173.95 |
| mean ±SD (g) | 210.57 ±164.86 | 210.57 ±164.86 | 210.57 ±164.86 | 210.57 ±164.86 | 210.57 ±164.86 | 210.57 ±164.86 | 210.57 ±164.86 | 210.57 ±164.86 | 210.57 ±164.86 |

© 2023 Bolte LA et al. *JAMA Oncology*.

| | | | | | | | | | |
|---|---|---|---|---|---|---|---|---|---|
| Patient ID | **MVCC001** | **MVCC006** | **MVCC011** | **MVCC012** | **MVCC018** | **MVCC024** | **MVCC041** | **MVCC052** | **MVCC054** |
| Centre | PRIMM-UK | PRIMM-UK | PRIMM-UK | PRIMM-UK | PRIMM-UK | PRIMM-UK | PRIMM-UK | PRIMM-UK | PRIMM-UK |
| Cheese (g) | 14.62 | 14.62 | 54.18 | 66.5 | 0 | 2.38 | 26.86 | 14.62 | 6.58 |
| median (g) | 14.62 | 14.62 | 14.62 | 14.62 | 14.62 | 14.62 | 14.62 | 14.62 | 14.62 |
| mean ±SD (g) | 14.98 ±13.51 | 14.98 ±13.51 | 14.98 ±13.51 | 14.98 ±13.51 | 14.98 ±13.51 | 14.98 ±13.51 | 14.98 ±13.51 | 14.98 ±13.51 | 14.98 ±13.51 |
| Milk (g) | 293 | 293 | 0 | 146 | 293 | 0 | 146 | 146 | 146 |
| median (g) | 293 | 293 | 293 | 293 | 293 | 293 | 293 | 293 | 293 |
| mean ±SD (g) | 246 ±140.27 | 246 ±140.27 | 246 ±140.27 | 246 ±140.27 | 246 ±140.27 | 246 ±140.27 | 246 ±140.27 | 246 ±140.27 | 246 ±140.27 |
| Yoghurt (g) | 0 | 17.64 | 0 | 26.46 | 0 | 0 | 8.82 | 126 | 8.82 |
| median (g) | 8.82 | 8.82 | 8.82 | 8.82 | 8.82 | 8.82 | 8.82 | 8.82 | 8.82 |
| mean ±SD (g) | 31.37 ±37.52 | 31.37 ±37.52 | 31.37 ±37.52 | 31.37 ±37.52 | 31.37 ±37.52 | 31.37 ±37.52 | 31.37 ±37.52 | 31.37 ±37.52 | 31.37 ±37.52 |
| Fruit juice (g) | 300 | 51.6 | 8.4 | 11.2 | 8.4 | 0 | 120 | 720 | 2.8 |
| median (g) | 8.4 | 8.4 | 8.4 | 8.4 | 8.4 | 8.4 | 8.4 | 8.4 | 8.4 |
| mean ±SD (g) | 47.78 ±114.41 | 47.78 ±114.41 | 47.78 ±114.41 | 47.78 ±114.41 | 47.78 ±114.41 | 47.78 ±114.41 | 47.78 ±114.41 | 47.78 ±114.41 | 47.78 ±114.41 |
| Sugar sweetened beverages (g) | 700 | 15.08 | 17.2 | 75.81 | 320.65 | 100 | 126 | 0 | 0 |
| median (g) | 17.2 | 17.2 | 17.2 | 17.2 | 17.2 | 17.2 | 17.2 | 17.2 | 17.2 |
| mean ±SD (g) | 86.58 ±162.28 | 86.58 ±162.28 | 86.58 ±162.28 | 86.58 ±162.28 | 86.58 ±162.28 | 86.58 ±162.28 | 86.58 ±162.28 | 86.58 ±162.28 | 86.58 ±162.28 |
| Tea (g) | 1140 | 1140 | 81.7 | 475 | 190 | 855 | 475 | 1140 | 936.7 |
| median (g) | 475 | 475 | 475 | 475 | 475 | 475 | 475 | 475 | 475 |
| mean ±SD (g) | 512.6 ±412.63 | 512.6 ±412.63 | 512.6 ±412.63 | 512.6 ±412.63 | 512.6 ±412.63 | 512.6 ±412.63 | 512.6 ±412.63 | 512.6 ±412.63 | 512.6 ±412.63 |
| Coffee (g) | 0 | 0 | 950 | 150.1 | 26.6 | 0 | 0 | 26.6 | 0 |
| median (g) | 150.1 | 150.1 | 150.1 | 150.1 | 150.1 | 150.1 | 150.1 | 150.1 | 150.1 |
| mean ±SD (g) | 265.72 ±333.68 | 265.72 ±333.68 | 265.72 ±333.68 | 265.72 ±333.68 | 265.72 ±333.68 | 265.72 ±333.68 | 265.72 ±333.68 | 265.72 ±333.68 | 265.72 ±333.68 |
| Nuts and seeds unsalted (g) | 0 | 0 | 15.8 | 0 | 0 | 0 | 0 | 0 | 1.4 |
| median (g) | 1.4 | 1.4 | 1.4 | 1.4 | 1.4 | 1.4 | 1.4 | 1.4 | 1.4 |
| mean ±SD (g) | 3.77 ±5.63 | 3.77 ±5.63 | 3.77 ±5.63 | 3.77 ±5.63 | 3.77 ±5.63 | 3.77 ±5.63 | 3.77 ±5.63 | 3.77 ±5.63 | 3.77 ±5.63 |

© 2023 Bolte LA et al. *JAMA Oncology*.

| | | | | | | | | | |
|---|---|---|---|---|---|---|---|---|---|
| Patient ID | **MVCC001** | **MVCC006** | **MVCC011** | **MVCC012** | **MVCC018** | **MVCC024** | **MVCC041** | **MVCC052** | **MVCC054** |
| Centre | PRIMM-UK | PRIMM-UK | PRIMM-UK | PRIMM-UK | PRIMM-UK | PRIMM-UK | PRIMM-UK | PRIMM-UK | PRIMM-UK |
| Potatoes (g) | 1383.15 | 233.36 | 95.33 | 0 | 242.66 | 52.78 | 0 | 233.36 | 17.64 |
| median (g) | 94.14 | 94.14 | 94.14 | 94.14 | 94.14 | 94.14 | 94.14 | 94.14 | 94.14 |
| mean ±SD (g) | 151.41 ±218.53 | 151.41 ±218.53 | 151.41 ±218.53 | 151.41 ±218.53 | 151.41 ±218.53 | 151.41 ±218.53 | 151.41 ±218.53 | 151.41 ±218.53 | 151.41 ±218.53 |
| Sauces and condiments (g) | 47.73 | 47.19 | 53.15 | 65.04 | 38.44 | 25.27 | 12.18 | 18.63 | 4.9 |
| median (g) | 18.63 | 18.63 | 18.63 | 18.63 | 18.63 | 18.63 | 18.63 | 18.63 | 18.63 |
| mean ±SD (g) | 23.27 ±15.22 | 23.27 ±15.22 | 23.27 ±15.22 | 23.27 ±15.22 | 23.27 ±15.22 | 23.27 ±15.22 | 23.27 ±15.22 | 23.27 ±15.22 | 23.27 ±15.22 |
| Soup (g) | 14 | 56 | 114 | 186 | 358 | 172 | 28 | 114 | 0 |
| median (g) | 28 | 28 | 28 | 28 | 28 | 28 | 28 | 28 | 28 |
| mean ±SD (g) | 43.49 ±64.49 | 43.49 ±64.49 | 43.49 ±64.49 | 43.49 ±64.49 | 43.49 ±64.49 | 43.49 ±64.49 | 43.49 ±64.49 | 43.49 ±64.49 | 43.49 ±64.49 |
| Vegetables (g) | 1267.05 | 281.62 | 322.63 | 554.6 | 374.03 | 75.54 | 49.72 | 212.95 | 67.3 |
| median (g) | 212.95 | 212.95 | 212.95 | 212.95 | 212.95 | 212.95 | 212.95 | 212.95 | 212.95 |
| mean ±SD (g) | 233.42 ±202.76 | 233.42 ±202.76 | 233.42 ±202.76 | 233.42 ±202.76 | 233.42 ±202.76 | 233.42 ±202.76 | 233.42 ±202.76 | 233.42 ±202.76 | 233.42 ±202.76 |
| Legumes and beans (g) | 65.94 | 166.53 | 124.11 | 78.82 | 125.65 | 17.64 | 44.73 | 51.94 | 4.9 |
| median (g) | 51.94 | 51.94 | 51.94 | 51.94 | 51.94 | 51.94 | 51.94 | 51.94 | 51.94 |
| mean ±SD (g) | 68.16 ±62.55 | 68.16 ±62.55 | 68.16 ±62.55 | 68.16 ±62.55 | 68.16 ±62.55 | 68.16 ±62.55 | 68.16 ±62.55 | 68.16 ±62.55 | 68.16 ±62.55 |

**eTable 11A. Description of outliers that corresponded to an implausible consumption in PRIM-UK.** Table shows the EI/BMR ratio and dietary intake of these individuals in comparison to the median and mean (SD) of the PRIMM-UK cohort. Abbreviations: BMI, body mass index; BMR, basal metabolic rate; EI/BMR, ratio of energy intake over basal metabolic rate; ffq, food frequency questionnaire; kcal, kilocalories; g, grams; principal component.

© 2023 Bolte LA et al. *JAMA Oncology*.

| eTable 11. Description of Outliers | | | |
|---|---|---|---|
| Patient ID | **OL 3925** | **POINTING-1-0029** | **POINTING-1-0033** |
| centre | PRIMM-NL | PRIMM-NL | PRIMM-NL |
| ANTHROPOMETRIC DATA | | | |
| age | 57 | 57 | 61 |
| sex | male | female | male |
| Weight | 70 | 83 | 93 |
| Height | 1.79 | 1.68 | 1.78 |
| BMI | 21.85 | 29.41 | 29.35 |
| BMR | 1539.22 | 1503.1 | 1823.47 |
| | | | |
| FOOD GROUP INTAKE | | | |
| Alcoholic beverages (freq/day) | 8 | 1 | 2 |
| median (freq/day) | 0 | 0 | 0 |
| mean±SD (freq/day) | 1.62 ±2.33 | 1.62 ±2.33 | 1.62 ±2.33 |
| Vegetables (freq/day) | 8 | 1 | 8 |
| median (freq/day) | 4 | 4 | 4 |
| mean±SD (freq/day) | 4.22 ±1.41 | 4.22 ±1.41 | 4.22 ±1.41 |
| Potatoes (freq/day) | 2 | 0 | 2 |
| median (freq/day) | 2 | 2 | 2 |
| mean±SD (freq/day) | 2.47 ±1.12 | 2.47 ±1.12 | 2.47 ±1.12 |
| Fruit (freq/day) | 0 | 0 | 1.5 |
| median (freq/day) | 1.5 | 1.5 | 1.5 |
| mean±SD (freq/day) | 1.52 ±0.63 | 1.52 ±0.63 | 1.52 ±0.63 |
| Fish (freq/day) | 0.07 | 0.4 | 0.07 |
| median (freq/day) | 0.1 | 0.1 | 0.1 |
| mean±SD (freq/day) | 0.13 ±0.10 | 0.13 ±0.10 | 0.13 ±0.10 |
| Meat (freq/day) | 2 | 1 | 0.5 |
| median (freq/day) | 1 | 1 | 1 |
| mean±SD (freq/day) | 1.1 ±0.42 | 1.1 ±0.42 | 1.1 ±0.42 |
| Cold cuts (freq/day) | 7 | 0 | 2 |
| median (freq/day) | 2 | 2 | 2 |
| mean±SD (freq/day) | 2.71 ±1.69 | 2.71 ±1.69 | 2.71 ±1.69 |
| Sauces (freq/day) | 2 | 1 | 2 |
| median (freq/day) | 2 | 2 | 2 |
| mean±SD (freq/day) | 1.6 ±0.58 | 1.6 ±0.58 | 1.6 ±0.58 |
| Soup (freq/day) | 0.1 | 0 | 0.8 |
| median (freq/day) | 0.1 | 0.1 | 0.1 |
| mean±SD (freq/day) | 0.23 ±0.22 | 0.23 ±0.22 | 0.23 ±0.22 |
| Cheese (freq/day) | 11.5 | 1.5 | 4.5 |
| median (freq/day) | 3.5 | 3.5 | 3.5 |

© 2023 Bolte LA et al. *JAMA Oncology.*

| | | | |
|---|---|---|---|
| mean±SD (freq/day) | 3.39 ±1.98 | 3.39 ±1.98 | 3.39 ±1.98 |

© 2023 Bolte LA et al. *JAMA Oncology*.

| Patient ID | OL 3925 | POINTING-1-0029 | POINTING-1-0033 |
|---|---|---|---|
| centre | **PRIMM-NL** | **PRIMM-NL** | **PRIMM-NL** |
| Milk (freq/day) | 1 | 6 | 1 |
| median (freq/day) | 1 | 1 | 1 |
| mean±SD (freq/day) | 1.58 ±1.12 | 1.58 ±1.12 | 1.58 ±1.12 |
| Savoury snacks and ready meals (freq/day) | 11.1 | 1.55 | 11 |
| median (freq/day) | 3.1 | 3.1 | 3.1 |
| mean±SD (freq/day) | 3.75 ±2.41 | 3.75 ±2.41 | 3.75 ±2.41 |
| Biscuits and cakes (freq/day) | 4 | 0.2 | 7.7 |
| median (freq/day) | 1.7 | 1.7 | 1.7 |
| mean±SD (freq/day) | 2.08 ±1.56 | 2.08 ±1.56 | 2.08 ±1.56 |
| Wholemeal bread (freq/day) | 4 | 0 | 5 |
| median (freq/day) | 2 | 2 | 2 |
| mean±SD (freq/day) | 2.72 ±1.76 | 2.72 ±1.76 | 2.72 ±1.76 |
| White bread (freq/day) | 0 | 0 | 0 |
| median (freq/day) | 0 | 0 | 0 |
| mean±SD (freq/day) | 0.61 ±1.07 | 0.61 ±1.07 | 0.61 ±1.07 |
| Fruit juice (freq/day) | 0.2 | 0.1 | 0.1 |
| median (freq/day) | 0.1 | 0.1 | 0.1 |
| mean±SD (freq/day) | 0.22 ±0.30 | 0.22 ±0.30 | 0.22 ±0.30 |
| Fats and oils (freq/day) | 0 | 1 | 1 |
| median (freq/day) | 1 | 1 | 1 |
| mean±SD (freq/day) | 1.42 ±0.58 | 1.42 ±0.58 | 1.42 ±0.58 |
| PRINCIPAL COMPONENT SCORES (DIETARY PATTERNS) | | | |
| PC 1 | 7.01 | -4.16 | 4.63 |
| median | 0.01 | 0.01 | 0.01 |
| mean±SD | 2.61e-16 ±1.84 | 2.61e-16 ±1.84 | 2.61e-16 ±1.84 |
| PC 2 | -2.28 | -0.74 | 1.14 |
| median | 0.3 | 0.3 | 0.3 |
| mean±SD | -2.3e-17 ±1.51 | -2.3e-17 ±1.51 | -2.3e-17 ±1.51 |
| PC 3 | 2.7 | 4.81 | -1.69 |
| median | -0.18 | -0.18 | -0.18 |
| mean±SD | -3.48e-16 ±1.43 | -3.48e-16 ±1.43 | -3.48e-16 ±1.43 |
| PC 4 | -0.23 | 2.33 | 3.37 |
| median | -0.02 | -0.02 | -0.02 |
| mean±SD | 1.49e-16 ±1.16 | 1.49e-16 ±1.16 | 1.49e-16 ±1.16 |
| PC 5 | -1.05 | 0.29 | -0.34 |
| median | -0.08 | -0.08 | -0.08 |
| mean±SD | 1.3e-16 ±1.11 | 1.3e-16 ±1.11 | 1.3e-16 ±1.11 |

© 2023 Bolte LA et al. *JAMA Oncology*.

eTable 11B. Description of outliers that corresponded to an implausible consumption in PRIMM-NL. Table shows dietary intake of these individuals in comparison to the median and mean (SD) of the PRIMMNL cohort. Abbreviations: BMI, body mass index, BMR, basal metabolic rate; freq/day, frequencies per day; PC; principal component; aMED, alternate Mediterranean diet score; original PDI, original plant-based diet index; further distinguished into: hPDI, healthy plant-based diet index, and uPDI, unhealthy plant-based diet index.

© 2023 Bolte LA et al. *JAMA Oncology*.

## eReferences


(1) Bingham SA, Welch AA, McTaggart A, Mulligan AA, Runswick SA, Luben R et al. Nutritional methods in the European Prospective Investigation of Cancer in Norfolk. *Public Health Nutr.* 2001;4(3):847-58. doi: 10.1079/phn2000102.

(2) van Lee L, Feskens EJ, Meijboom S, Hooft van Huysduynen EJ, van't Veer P, de Vries JH et al. Evaluation of a screener to assess diet quality in the Netherlands. *Br J Nutr.* 2016;115(3):517-26. doi: 10.1017/S0007114515004705.

(3) Mulligan AA, Luben RN, Bhaniani A, Parry-Smith DJ, O'Connor L, Khawaja AP, Forouhi NG, Khaw KT; EPIC-Norfolk FFQ Study. A new tool for converting food frequency questionnaire data into nutrient and food group values: FETA research methods and availability. *BMJ Open*. 2014;4(3):e004503. doi:10.1136/bmjopen-2013-004503.

(4) Banna JC, McCrory MA, Fialkowski MK, Boushey C. Examining plausibility of self-reported energy intake data: considerations for method selection. *Front Nutr.* 2017;25(4):45. doi: 10.3389/fnut.2017.00045.

(5) National Institute for Public Health and the Environment (RIVM). Dutch Food Composition Database. https://www.rivm.nl/en/dutch-food-composition-database. Accessed February 12, 2022.

(6) Public Health England. McCance and Widdowson's The Composition of Foods Integrated Dataset. https://assets.publishing.service.gov.uk/government/uploads/system/uploads/attachment_data/file/971021/McCance_and_Widdowsons_Composition_of_Foods_integrated_dataset_2021.pdf. Accessed February 12, 2022.

(7) Fung TT, McCullough ML, Newby PK, Manson JE, Meigs JB, Rifai N, Willett WC, Hu FB. Diet-quality scores and plasma concentrations of markers of inflammation and endothelial dysfunction. *Am J Clin Nutr.* 2005;82(1):163-73. doi: 10.1093/ajcn.82.1.163.

(8) Bowyer RCE, Jackson MA, Pallister T, Skinner J, Spector TD, Welch AA, Steves CJ. Use of dietary indices to control for diet in human gut microbiota studies. *Microbiome.* 2018;6(1):77. doi: 10.1186/s40168-018-0455-y.

(9) Satija A, Bhupathiraju SN, Spiegelman D, Chiuve SE, Manson JE, Willett W et al. Healthful and Unhealthful Plant-Based Diets and the Risk of Coronary Heart Disease in U.S. Adults. *J Am Coll Cardiol.* 2017;70(4):411-422. doi: 10.1016/j.jacc.2017.05.047.

(10) Tucker KL. Dietary patterns, approaches, and multicultural perspective. *Appl Physiol Nutr Metab.* 2010;35(2):211-8. doi: 10.1139/H10-010.


© 2023 Bolte LA et al. *JAMA Oncology.*

(11) Wood SN. *Generalized Additive Models: An Introduction with R* (*2nd edition)*. New York, NY: Chapman and Hall/CRC; 2017. doi: 10.1201/9781315370279.

(12) Sivan A, Corrales L, Hubert N, Williams JB, Aquino-Michaels K, Earley ZM et al. Commensal Bifidobacterium promotes antitumor immunity and facilitates anti-PD-L1 efficacy. *Science.* 2015;350(6264):1084-9. doi: 10.1126/science.aac4255.

(13) Yuan X, Duan Y, Xiao Y, Sun K, Qi Y, Zhang Y, Ahmed Z et al. Vitamin E Enhances Cancer Immunotherapy by Reinvigorating Dendritic Cells via Targeting Checkpoint SHP1. *Cancer Discov.* 2022;12(7):1742–1759. doi: 10.1158/2159-8290.CD-21-0900.

(14) Magrì A, Germano G, Lorenzato A, Lamba S, Chilà R, Montone M et al. High-dose vitamin C enhances cancer immunotherapy. *Sci Transl Med.* 2020;12(532):eaay8707. doi: 10.1126/scitranslmed.aay8707.

(15) Huang J, Liu D, Wang Y, Liu L, Li J, Yuan J et al. Ginseng polysaccharides alter the gut microbiota and kynurenine/tryptophan ratio, potentiating the antitumour effect of antiprogrammed cell death 1/programmed cell death ligand 1 (anti-PD-1/PD-L1) immunotherapy. *Gut.* 2022;71(4):734-745. doi: 10.1136/gutjnl-2020-321031.

(16) Wang F, Yin Q, Chen L, Davis MM. *Bifidobacterium* can mitigate intestinal immunopathology in the context of CTLA-4 blockade. *Proc Natl Acad Sci U S A*. 2018;115(1):157-161. doi: 10.1073/pnas.1712901115.

(17) Vétizou M, Pitt JM, Daillère R, Lepage P, Waldschmitt N, Flament C et al. Anticancer immunotherapy by CTLA-4 blockade relies on the gut microbiota. *Science.* 2015;350(6264):1079-84. doi: 10.1126/science.aad1329.

(18) Ferrere G, Tidjani Alou M, Liu P, Goubet AG, Fidelle M, Kepp O et al. Ketogenic diet and ketone bodies enhance the anticancer effects of PD-1 blockade. *JCI Insight.* 2021;6(2):e145207. doi: 10.1172/jci.

(19) Spencer CN, McQuade JL, Gopalakrishnan V, McCulloch JA, Vetizou M, Cogdill AP et al. Dietary fiber and probiotics influence the gut microbiome and melanoma immunotherapy response. *Science.* 2021;374(6575):1632-1640. doi: 10.1126/science.aaz7015.

(20) Nomura M, Nagatomo R, Doi K, Shimizu J, Baba K, Saito T et al. Association of short-chain fatty acids in the gut microbiome with clinical response to treatment with nivolumab or pembrolizumab in patients with solid cancer tumors. *JAMA Netw Open*. 2020;3(4):e202895. doi: 10.1001/jamanetworkopen.2020.2895.

(21) Simpson RC, Shanahan ER, Batten M, Reijers ILM, Read M, Silva IP, et al. Diet-driven microbial ecology underpins associations between cancer immunotherapy outcomes and the gut microbiome. *Nat Med* 2022. Epub ahead of print. doi: 10.1038/s41591-022-01965-2.

© 2023 Bolte LA et al. *JAMA Oncology*.
