# Supplementary material for: Association of a Mediterranean Diet With Outcomes for Patients Treated With Immune Checkpoint Blockade for Advanced Melanoma
Source: JAMA Oncol. 2023 Feb 16;9(5):705–9. doi: 10.1001/jamaoncol.2022.7753 (PMC9936383; doi:10.1001/jamaoncol.2022.7753)
Supplement: Supplement 2. — Data sharing statement [file jamaoncol-e227753-s002.pdf]

Data Sharing Statement

Bolte. Association of a Mediterranean Diet With Outcomes for Patients Treated With Immune Checkpoint Blockade for Advanced Melanoma. *JAMA Oncol*. Published February 16, 2023. doi:10.1001/jamaoncol.2022.7753

Data
Data available: Yes
Data types: Deidentified participant data
How to access data: All relevant data supporting the key findings of this study are available within the article and the supplementary files. Other data are available from the corresponding author upon reasonable requests (r.k.weersma@umcg.nl)
When available: With publication

Supporting Documents
Document types: Statistical/analytic code
How to access documents: All statistical analysis scripts are written in R and can be found here: https://github.com/WeersmaLabIBD.
When available: With publication

Additional Information
Who can access the data: All relevant data supporting the key findings of this study are available within the article and the supplementary files. Other data are available from the corresponding author upon reasonable requests.
Types of analyses: Joining of datasets e.g. in a meta-analysis or for replication in independent cohorts.
Mechanisms of data availability: All relevant data supporting the key findings of this study are available within the article and the supplementary files. Other data are available from the corresponding author upon reasonable requests.
